# KYNU as a Biomarker of Tumor-Associated Macrophages and Correlates with Immunosuppressive Microenvironment and Poor Prognosis in Gastric Cancer

**DOI:** 10.1155/2023/4662480

**Published:** 2023-11-02

**Authors:** Kaiyu Shen, Binyu Chen, Liu Yang, Wencang Gao

**Affiliations:** ^1^The Second Clinical Medical College of Zhejiang Chinese Medical University, Hangzhou 310053, China; ^2^Department of Oncology, The Second Affiliated Hospital, Zhejiang Chinese Medical University, Hangzhou 310005, China

## Abstract

**Background:**

Kynureninase (KYNU) is a potential prognostic marker for various tumor types. However, no reports on the biological effects and prognostic value of KYNU in gastric cancer (GC) exist.

**Methods:**

GC-associated single-cell RNA sequencing and bulk RNA sequencing (bulk-seq) data were obtained from the Gene Expression Omnibus and The Cancer Genome Atlas databases, respectively. The differential expression of KYNU between GC and normal gastric tissues was first analyzed based on the bulk-seq data, followed by an exploration of the relationship between KYNU and various clinicopathological features. The Kaplan–Meier survival and Cox regression analyses were performed to determine the prognostic value of KYNU. The relationship between KYNU expression and immune cell infiltration and immune checkpoints was also explored. The biological function of KYNU was further examined at the single-cell level, and in vitro experiments were performed to examine the effect of KYNU on GC cell proliferation and invasion.

**Results:**

KYNU expression was significantly elevated in GC samples. Clinical features and survival analysis indicated that high KYNU expression was associated with poor clinical phenotypes and prognosis, whereas Cox analysis showed that KYNU was an independent risk factor for patients with GC. Notably, high expression of KYNU induced a poor immune microenvironment and contributed to the upregulation of immune checkpoints. KYNU-overexpressing macrophages drove GC progression through unique ligand-receptor pairs and transcription factors and were associated with adverse clinical phenotypes in GC. KYNU was overexpressed in GC cells in vitro, and KYNU knockout significantly inhibited GC cell proliferation and invasion.

**Conclusion:**

High KYNU expression promotes an adverse immune microenvironment and low survival rates in GC. KYNU and KYNU-related macrophages may serve as novel molecular targets in the treatment of GC.

## 1. Introduction

Gastric cancer (GC) is one of the most prevalent malignant tumor types that affects the digestive system. The progression from normal gastric epithelial tissue to GC involves atrophy, intestinal metaplasia, and intraepithelial neoplasia. While increased public awareness and improved dietary structures have somewhat reduced the incidence and mortality rates of GC, the prognosis for advanced GC remains poor, primarily due to the fact that the symptoms for the majority of patients can be concealed [1]. Genes, proteins, and various molecular metabolic products contribute to biological activities. As a result, transcriptomic-based biomarkers have gradually become prominent in cancer detection.

In recent years, the role of tryptophan metabolism in the tumor microenvironment (TME) has attracted considerable attention. The role of tryptophan metabolites in driving tumor cell invasion and migration has been confirmed in breast cancer [2] and lung cancer [3]. Additionally, tryptophan metabolites induce immunosuppressive cells, such as the infiltration of regulatory T cells and upregulation of immune checkpoints, playing a role in immune evasion [4, 5]. Key enzymes of tryptophan metabolism, such as indoleamine-2,3-dioxygenase 1, tryptophan-2,3-dioxygenase, and kynurenine monooxygenase, play essential roles in pathophysiological processes and have been found to be overexpressed in melanoma, ovarian cancer, breast cancer, and colon cancer [6].

Kynureninase (KYNU) is another key enzyme in the tryptophan metabolism pathway, involved in the production of two tryptophan metabolites: 3-hydroxyanthranilic and anthranilic acid [7]. Among these, 3-hydroxyanthranilic has been found to be closely related to lower survival in non-small cell lung cancer and renal cell carcinoma [8, 9]. In squamous carcinoma, KYNU overexpression promotes cancer cell proliferation and invasion through the PI3K/AKT pathway [10]. Zhou et al. confirmed through in vitro experiments that silencing KYNU significantly inhibited the tumor-promoting effects of indoleamine-2,3-dioxygenase on prostate cancer cells [11]. However, there are few reports on whether KYNU is a prognostic biomarker of GC. Currently, the application of bioinformatics has opened up new avenues for research on disease mechanisms and treatment methods, especially the development of single-cell RNA sequencing (scRNA-seq) technology, which not only enhances our understanding of biomarkers at the cellular subpopulation level but also provides a new theoretical basis for the research and development of drug targets [12]. Therefore, in this study, we used bioinformatics and bulk-seq data to analyze the diagnostic and prognostic value of KYNU in GC. In addition, we explored the biological characteristics of KYNU at the single-cell level in GC and investigated the effects of KYNU gene silencing on GC cell proliferation and invasion. This study investigated the potential of KYNU as a prognostic biomarker and target for antitumor treatment of GC.

## 2. Materials and Methods

### 2.1. Data Acquisition

Transcriptomic and paired clinical data containing 32 normal and 375 GC samples were obtained from The Cancer Genome Atlas (TCGA) database (https://cancergenome.nih.gov/),

Among them, GC samples were used for further constructing the nomogram. We excluded GC samples with incomplete clinical information that met the following criteria: (1) absence of KYNU gene expression; (2) lack of clinical features including age, sex, pathological grade, and tumor stage; and (3) missing survival status and survival time data. Ultimately, a total of 317 GC samples with complete clinical information and matched transcriptome data were obtained. The TCGA-stomach adenocarcinoma (STAD) cohort was stratified into a training set and a validation set using a 6 : 4 ratio. This study employed standard transcriptome data of normal gastric tissue and GC tissue retrieved from the Gene Expression Omnibus (GEO) database (http://www.ncbi.nlm.nih.gov/geo). Specifically, datasets GSE27342 (normal = 80, tumor = 80), GSE29272 (normal = 134, tumor = 134), and GSE65801 (normal = 32, tumor = 32) were utilized for external validation of KYNU gene expression. To convert probe-level expression values (probe ID) to their respective gene symbols, annotation files were used. If multiple probes were found to match the same gene, the average value was calculated to determine the gene's expression level. In addition, we obtained 10 GC samples from GSE167297, including the scRNA-seq data from five superficial GC, five deep GC, and four paracancerous samples [13].

### 2.2. KYNU Expression Analysis

Based on the “limma” and “ggplot2” packages, we extracted the expression profiles of KYNU in normal gastric tissue and GC samples from the GEO dataset. The “normalizeBetweenArrays” function was used to standardize the data, and the differential expression of KYNU was calculated using the “limma” package and “ggpubr” package in both the TCGA dataset and GEO dataset. Additionally, the “ggpaired” function was used to compare the paired differential expression of KYNU between GC and paired normal tissues. We performed calculations to determine the Log_2_ Fold-Change (FC) values of KYNU in both GC and normal gastric tissues. Subsequently, the “intersect” function was used to organize the GC samples with complete clinical information and KYNU expression. After converting the clinical information into factors using the “factor” function, we compared the expression of KYNU in different clinical features using the Kruskal-Wallis test. In addition, we divided KYNU into high and low-expression groups based on the median expression value and compared its relationship with clinical features.

### 2.3. Prognostic Implications of KYNU

The overall survival (OS) curves of KYNU expression in GC samples were obtained from the Gene Expression Profiling Interactive Analysis (GEPIA) web server (http://gepia.cancer-pku.cn/index.html). By consolidating the clinical information on GC in the TCGA database, univariate and multivariate Cox analyses were performed to determine whether KYNU was an independent prognostic factor, and the KYNU-nomogram was constructed to predict the one-, three-, and five-year survival rates of patients with GC.

### 2.4. Functional Enrichment Analysis

Gene set enrichment analysis (GSEA) was performed to assist in the interpretation of data from gene expression profiles by setting specific functional gene sets [14]. We employed the GSEA software (v. 4.1.0) to analyze the signaling pathways in which KYNU may be involved in the TCGA–STAD cohort, with a permutation test parameter of 1000, and gene set parameters of “h.all.v2022.1.Hs.symbols.gmt” and “c2.cp.kegg.v2022.1.Hs.symbols.gmt.” The threshold for statistical significance was set at NOM *P* value < 0.05.

### 2.5. Analysis of KYNU and the Immune Microenvironment and Immune Checkpoint Expression

To investigate the influence of KYNU on tumor-infiltrating immune cells (TICs), we used the single-sample GSEA (ssGSEA) algorithm to calculate the abundance of TICs and immune-related pathway scores in each GC sample from the TCGA–STAD cohort. The limma package was used to identify the differences in the level of TICs and immune function activity between the high KYNU and low KYNU groups. Subsequently, the differential expressions of various immune checkpoints were compared between the high and low KYNU groups.

### 2.6. scRNA-Seq Data Preprocessing

scRNA-seq data of GC and paracancerous samples were processed using the Seurat package in R (version 4.1.0) software [15]. Batch effects between the samples were removed using the Harmony package. Low-quality cells were discarded based on the criteria of high (> 15%) mitochondrial DNA percentage and number of expressed genes < 500. A total of 2000 hypervariable genes were then selected for principal component analysis (PCA), and cell subpopulations were clustered using the *t*-distributed stochastic neighbor embedding (t-SNE) algorithm (resolution 0.5). The “FindAllMarkers” function was used to detect the differentially expressed genes (DEGs) in each cell cluster. With the assistance of the CellMarker database [16], the cell types were annotated, and KYNU-expressing cell subpopulations were named KYNU-related cells. Subsequently, the AUCell package was employed to explore the pathway activity within the cell subpopulations. The PercentageFeatureSet function was used to analyze the expression percentage of KYNU in KYNU-related cells, with KYNU(+)- and KYNU(-)-related cells defined as cells with KYNU expression percentages > 0 and = 0, respectively. The “FindAllMarkers” function was used to find markers by comparing each cluster with all others; different genes between two identities were identified using the FindMarkers function. Referring to the methods of Qiao et al. [17], the FindMarkers function (parameter settings: min.pct = 0.1, logfc.threshold = 0.3) was used to identify DEGs between KYNU(+)-related cells and KYNU(-)-related cells.

### 2.7. Trajectory Analysis

The Monocle2 package was employed to analyze the developmental trajectory of KYNU-related cells over the course of GC progression [18]. To further investigate KYNU-related biological mechanisms at the single-cell level in GC, we calculated the gene clusters along the cell development trajectory and performed enrichment analysis on gene clusters with consistent KYNU expression patterns.

### 2.8. Cell-Cell Communication Analysis

To investigate the impact of KYNU on the expression of ligand-receptor pairs at the cellular level, we used the CellPhone database (CellPhoneDB) [19] to infer the potential interaction strength between KYNU-related cells and other cell subpopulations. Subsequently, the CellChat package [20] was employed to provide a detailed analysis of the differences in ligand-receptor pairs enriched in both KYNU(+)-related cells and KYNU(-)-related cells, with the CellChat database parameters set as “Secreted Signaling,” “ECM-Receptor,” and “Cell-Cell Contact.”

### 2.9. Single-Cell Regulatory Network Inference and Clustering (SCENIC) Analysis

We randomly selected the gene expression profiles of 1000 KYNU-related cells to infer the transcriptional regulatory network of KYNU(+)-related cells using the SCENIC package [21]. The GENIE3 function was then applied to construct the coexpression modules of transcription factors (TFs), and the RcisTarget package was employed to identify TFs and their regulatory targets. Evaluation of specific TFs in KYNU(+)-related cells was then performed using the regulon specificity score (RSS). In addition, we employed the AUCell package to calculate the activity of TFs in cell subpopulations.

### 2.10. Determination of the Relationship between KYNU-Related Cells and Clinical Phenotypes Based on the Scissor Algorithm

The Scissor algorithm uses phenotypic information to guide the identification of key cell subpopulations [22]. We used the Scissor package to explore the association between KYNU-related cells and specific clinical phenotypes based on the survival status in the TCGA–STAD cohort (Dead and Alive), the “Tumor” and “Normal” phenotypes, significant indicators from previous analyses of clinical characteristics, bulk-seq data, and scRNA-seq from KYNU-related cells. We first selected 500 KYNU(+)-related cells with the highest KYNU expression and 500 KYNU(-)-related cells with the lowest KYNU expression. Then, with reference to the methods described in the original paper for the Scissor algorithm [22], the clinical phenotypes were set as binary values (0 and 1), and the Scissor function was used to divide the KYNU-related cells into three subpopulations: Scissor+, Scissor-, and Background cells. Among which, a phenotype with an indicator value of “1” was positively correlated with Scissor+ cells, and “0” was positively correlated with Scissor- cells, while Background cells were not correlated with the phenotype. Finally, chi-squared test was performed to analyze the differences in the proportions of KYNU(+)-related cells and KYNU(-)-related cells for each phenotype.

### 2.11. Cell Culture and Transfection

One normal gastric cell line (GES-1) and three GC cell lines (MKN-45, BGC-823, and Hs-746T) were purchased from Hangzhou Frieden Biotechnology Co., Ltd. The MKN-45, BGC-823, and Hs-746T cells were cultured in RPMI-1640 medium, whereas the GES-1 cells were cultured in Dulbecco's modified Eagle's medium (DMEM) containing 10% fetal bovine serum. All cells were cultured at 37°C under 5% CO_2_. Silencing of KYNU was achieved through the human target gene KYNU short hairpin RNA (shRNA) (purchased from GenePharma Co., Ltd, Shanghai, China) with the following sequences: KYNU-shRNA-1: 5′-TGAATGCTTTGACTGTAAATT-3′, KYNU-shRNA-2: 5′-CTGTGGTTATTTCAGTATTAT-3′, and KYNU-shRNA-3: 5′-TGGTGTTCCTACAAGTATTTA-3′. Lipofectamine 3000 (Invitrogen, USA) was used to transfect BGC-823 cells, and those infected for 72 h were used for subsequent analysis.

### 2.12. Quantitative Reverse Transcription PCR (qRT-PCR)

To quantify KYNU expression in the normal gastric cell line and GC cell lines, total RNA was extracted from GES-1 and the remaining three GC cell lines using the TRIzol Reagent. To initiate the process, mix 1 *μ*l of cells with 99 *μ*l of diethylpyrocarbonate water. Subsequently, utilize the NanoDrop-2000 (Thermo Fisher Scientific, USA) to determine the values of OD260 nm, OD280 nm, and OD260/OD280 nm for the sample. If the OD260/OD280 nm ratio fell within the range of 1.8 to 2.0, the extracted RNA was deemed suitable for the subsequent reverse transcription step. Ratios of OD260/OD280 nm greater than 2.1 or less than 1.8 were indicative of RNA contamination. Thereafter, qRT-PCR was performed using SYBR Premix Ex Taq according to the manufacturer's protocol; *β*-actin was used as an internal control. The expression level of KYNU was quantified using the 2^−ΔΔCT^ method. The primers for *β*-actin were F: 5′-TGGCACCCAGCACAATGAA-3′ and R: 5′-CTAAGTCATAGTCCGCCTAGAAGCA-3′, and those for KYNU were F: 5′-CCTGCGAGATCGGAGTTCTT-3′ and R: 5′-GGTCTCTCTAAAGCTCTTGTCCT-3′.

### 2.13. Cell Proliferation Assays

Referring to previous studies' experimental methods [23–25], the BGC-823 cell line was used for subsequent analyses. To evaluate the proliferative capacity of GC cells after KYNU silencing, BGC-823 cells were transfected with KYNU-shRNA and NC-shRNA. Transfected cells were incubated at 37°C and 5% CO_2_. Cell proliferation was evaluated using the cell counting kit-8 (CCK-8, Dojindo, Tokyo, Japan) assay as per manufacturer's instructions, and the absorbance at 450 nm was measured at 0, 24, 48, and 72 h posttransfection.

### 2.14. Wound Healing Test

The transfected BGC-823 cells mentioned above were inoculated in 6-well plates at a density of 1 × 10^5^ cells/well. After the cells adhered and reached 80%–100% confluence, they were wounded with a 2 *μ*L sterile pipette tip, washed three times with phosphate-buffered saline (PBS), and then cultured in fresh complete medium for 48 h. The cultures were photographed at 0, 12, 24, and 48 h under a microscope at 100x magnification.

### 2.15. Cell Invasion Assays

The cell invasion ability of BGC-823 cells was evaluated using a transwell chamber (Corning, New York, USA). The BGC-823 cells transfected with KYNU-shRNA and NC-shRNA were placed in the upper chamber, whereas the complete medium was placed in the lower chamber. After incubation at 37°C with 5% CO_2_ for 48 h, the upper layer of noninvaded cells was removed with cotton swabs, and the BGC-823 cells were fixed and stained with 4% paraformaldehyde and 0.1% crystal violet, respectively. The cells were observed under a microscope at 400x magnification and analyzed using ImageJ software (version 1.8.0.112).

### 2.16. Colony Formation Assays

The NC-shRNA- and KYNU-shRNA-transfected BGC-823 cells were cultured for 2 weeks, and the density was set as 500 cells/well. The cells were fixed with 4% paraformaldehyde and stained with crystal violet, followed by quantitative analysis using ImageJ software (version 1.8.0.112).

### 2.17. Statistical Analysis

All statistical analyses were performed using R software (version 4.1.0). The Wilcoxon and Kruskal–Wallis tests were used to compare the differences between two groups and multiple groups, respectively. The Kaplan–Meier method and log-rank test were used to determine the differences in OS between the high- and low-KYNU expression groups. The univariate and multivariate Cox regression analyses were used to determine the independent prognostic factors. Spearman's rank correlation was used for correlation analysis. The graphs were plotted using R (version 4.1.0) and GraphPad Prism (version 7.0) software. All experiments were repeated three times. Unless stated otherwise, the differences were considered statistically significant at P < 0.05. To mitigate the influence of random variation, the experiment is conducted in triplicate.

## 3. Results

### 3.1. KYNU Expression in GC

**Figure 1:**
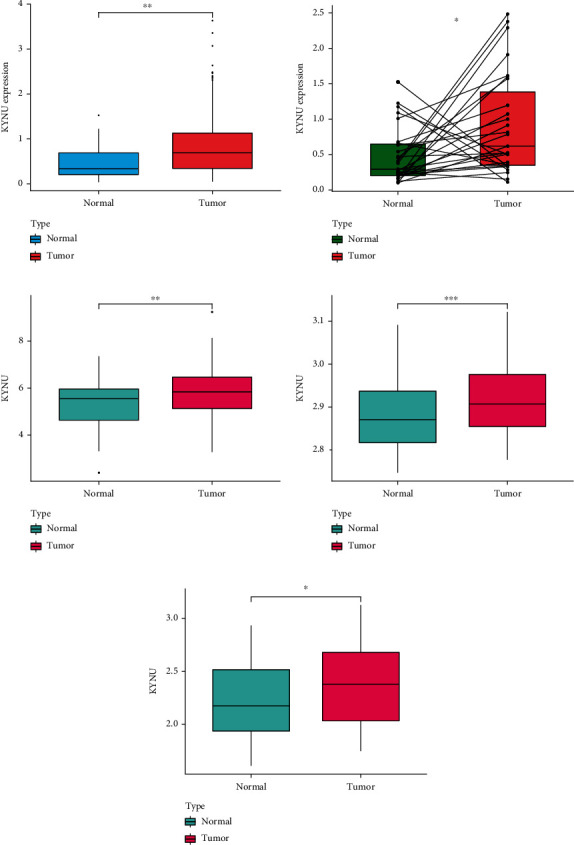
Differential expression analysis of KYNU. (a) Analysis of KYNU differential expression in normal and GC tissues based on TCGA–STAD cohort. (b) Differential expression of KYNU between GC and paired normal tissues. (c–e) Differential expression of KYNU in the GSE27342, GSE29272, and GSE65801 cohorts (^∗^*P* < 0.05, ^∗∗^*P* < 0.01, and ^∗∗∗^*P* < 0.001).

**Figure 2:**
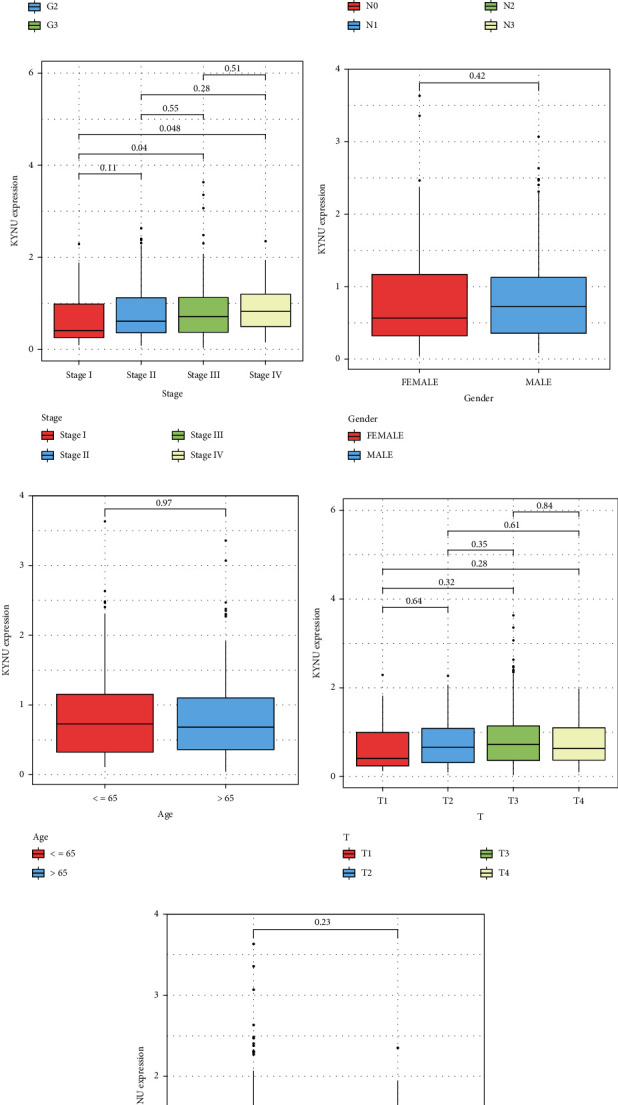
Analysis of clinical features. Differential analysis of KYNU expression with respect to (a) tumor grade, (b) N stage, (c) TNM stage, (d) sex, (e) age, (f) T stage, and (g) distant metastasis.

Analysis of the TCGA–STAD dataset indicated that KYNU expression was significantly higher in GC than in normal gastric tissue (*P* < 0.01; Log_2_FC = 0.316) (Figure 1(a)) and paired normal tissue (*P* < 0.05) (Figure 1(b)). These findings were verified in the three datasets: GSE27342 (*P* < 0.01; Log_2_FC = 0.517), GSE29272 (*P* < 0.001; Log_2_FC = 0.173), and GSE65801 (*P* < 0.05; Log_2_FC = 0.735) (Figures 1(c)–1(e)). Analysis of clinical features indicated that KYNU expression was elevated in high-grade GC (*P* < 0.01) (Figure 2(a)). In addition, KYNU was associated with the extent of lymph node metastasis (N). KYNU expression was higher in the N3 stage than in the N0 stage (*P* < 0.05) (Figure 2(b)). Regarding tumor node metastasis (TNM) staging, KYNU expression was higher in stage III and stage IV than in stage I (*P* < 0.05) (Figure 2(c)). However, KYNU expression was not associated with sex, age, tumor spread (T), or distant metastasis (M) (*P* > 0.05) (Figures 2(d)–2(g)). In addition, we further compared the clinical features between 158 GC samples with high KYNU expression and 159 GC samples with low KYNU expression in Supplementary Table S1.

### 3.2. Associations between KYNU Expression and the Survival Prognosis of Patients with GC

**Figure 3:**
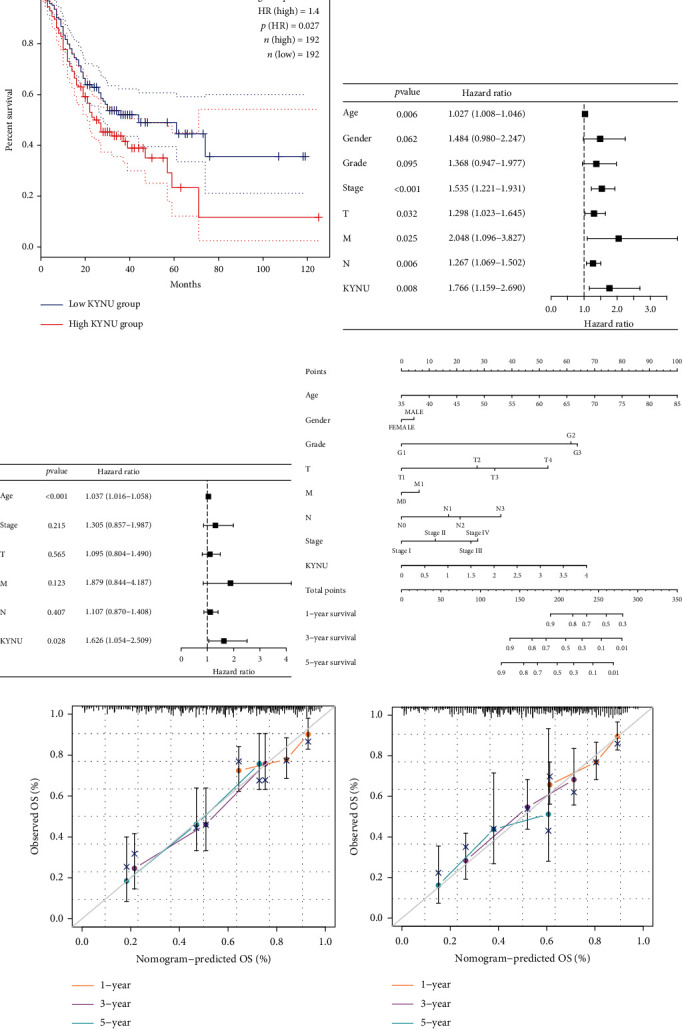
Survival analysis and prognostic value of KYNU. (a) OS of patients with GC with high and low KYNU expression in the GEPIA database. (b) Univariate and (c) multivariate Cox regression analyses of KYNU and other clinical features. (d) KYNU-nomogram constructed by integrating KYNU with various clinical features. (e, f) The calibration curves of the KYNU-nomogram in the training and validation sets.

To assess the prognostic value of KYNU in GC, we employed the GEPIA database to perform the Kaplan–Meier survival analysis. High KYNU expression was positively correlated with poor OS in GC (*P* < 0.05) (Figure 3(a)). Univariate Cox analysis also suggested that high KYNU expression was associated with a high hazard ratio (HR) (HR = 1.766, 95% confidence interval (CI): 1.159–2.690, *P* = 0.008) (Figure 3(b)). Subsequent multivariate Cox analysis confirmed that KYNU (HR = 1.626, 95% CI: 1.054–2.509, *P* = 0.028) and age (HR = 1.037, 95% CI: 1.016–1.058, *P* < 0.001) were independent prognostic factors (Figure 3(c)). The TCGA cohort was divided into a training set (*n* = 191) and a validation set (*n* = 126) in a ratio of 6 : 4. In the training set, we developed a KYNU-nomogram (Figure 3(d)) to predict 1-, 3-, and 5-year outcomes by incorporating clinical features including age, gender, grade, stage, T, N, and M staging, and KYNU expression. Subsequently, the calibration curves illustrated the strong predictive performance of the KYNU-nomogram in both the training and validation sets, as showcased in Figures 3(e) and 3(f).

### 3.3. Functional Analysis of KYNU

**Figure 4:**
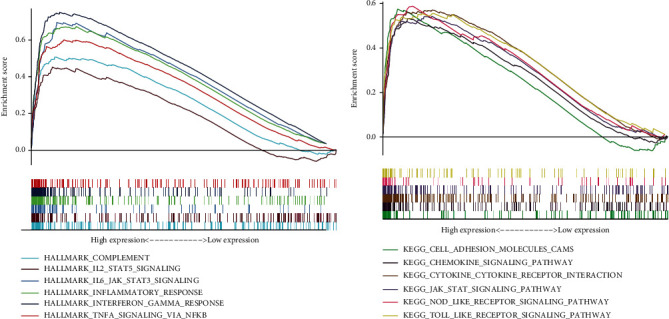
Functional enrichment analysis of KYNU. (a, b) Functional enrichment analysis based on the HALLMARK gene set and KEGG gene set.

Through GSEA, we explored the signaling pathways in which KYNU may be involved based on two gene sets. The results showed that COMPLEMENT, IL2/STAT5 signaling, IL-6/JAK/STAT3 signaling, inflammatory response, TNFA signaling via NFkB, chemokine signaling pathway, NOD-like receptor signaling pathway, and Toll-like receptor signaling pathway were involved in enrichment in the high KYNU group (Figures 4(a) and 4(b)). Thus, high KYNU expression may promote the progression of GC via these pathways.

### 3.4. KYNU Expression Is Correlated with TICs and Immune Checkpoints

**Figure 5:**
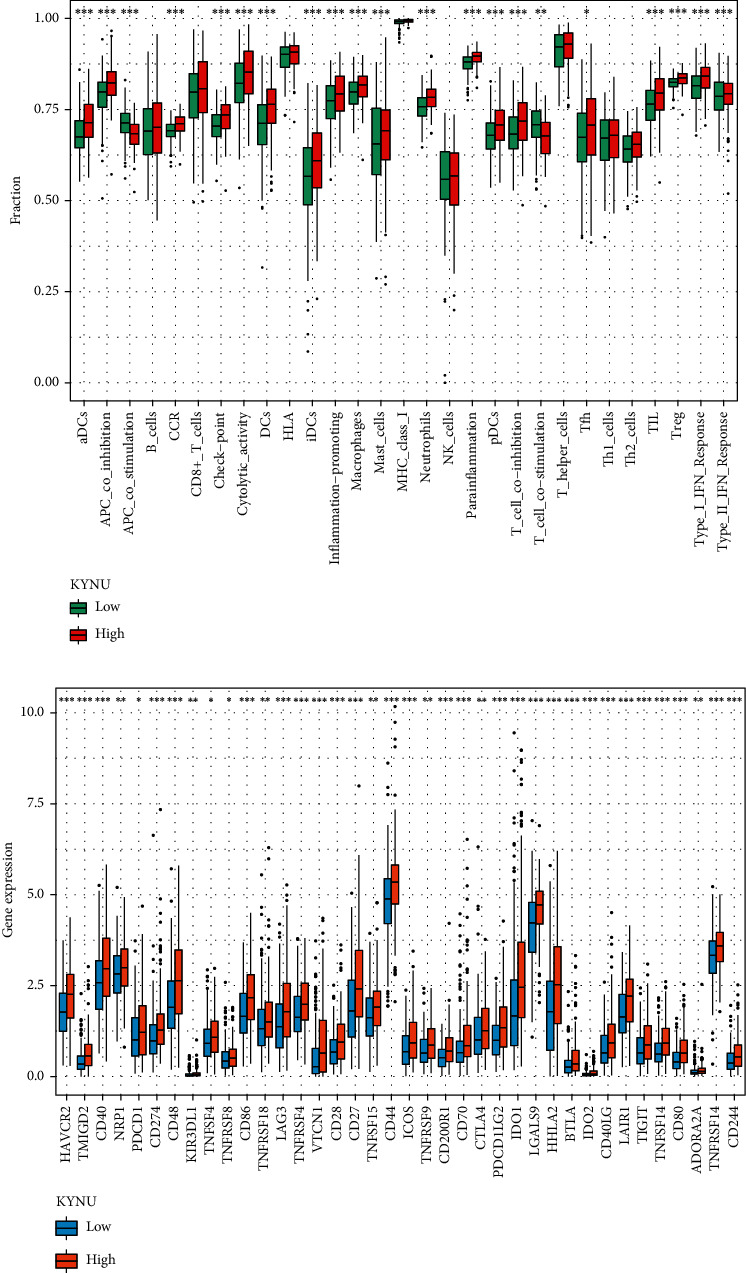
Relationship between KYNU expression and TICs and immune checkpoints. (a) ssGSEA analysis of KYNU group. (b) Expression of immune checkpoints in the high and low KYNU groups (^∗^*P* < 0.05, ^∗∗^*P* < 0.01, and ^∗∗∗^*P* < 0.001).

KYNU is thought to be involved in the regulation of immune responses [26]. Thus, we examined the relationship between KYNU expression and TICs. As shown in Figure 5(a), the degrees of infiltration of immune cells such as CD8 T cells (*P* < 0.001), mast cells (*P* < 0.001), macrophages (*P* < 0.001), neutrophils (*P* < 0.001), plasmacytoid dendritic cells (pDCs) (*P* < 0.001), and regulatory T cells (Tregs) (*P* < 0.001) were significantly increased in GC patients with high KYNU expression. Notably, immune-related pathways such as chemokine receptor (CCR) (*P* < 0.001), parainflammation (*P* < 0.001), type 1 IFN (*P* < 0.001), antigen-presenting cell (APC) coinhibition (*P* < 0.001), and T cell coinhibition (*P* < 0.001) were active in the KYNU high-expression group. Furthermore, the expressions of checkpoints, including programmed cell death protein 1 (PDCD1) (*P* < 0.05), cytotoxic T-lymphocyte associated protein 4 (CTLA4) (*P* < 0.01), lymphocyte-activation protein 3 (LAG3) (*P* < 0.001), hepatitis A virus cellular receptor 2 (HAVCR2) (*P* < 0.001), and T cell immunoreceptor with Ig and ITIM domains (TIGIT) (*P* < 0.001), were significantly elevated in the high KYNU expression group (Figure 5(b)). These results suggest that KYNU expression is involved in the regulation of the GC immune microenvironment.

### 3.5. Expression of KYNU at the Single-Cell Level

**Figure 6:**
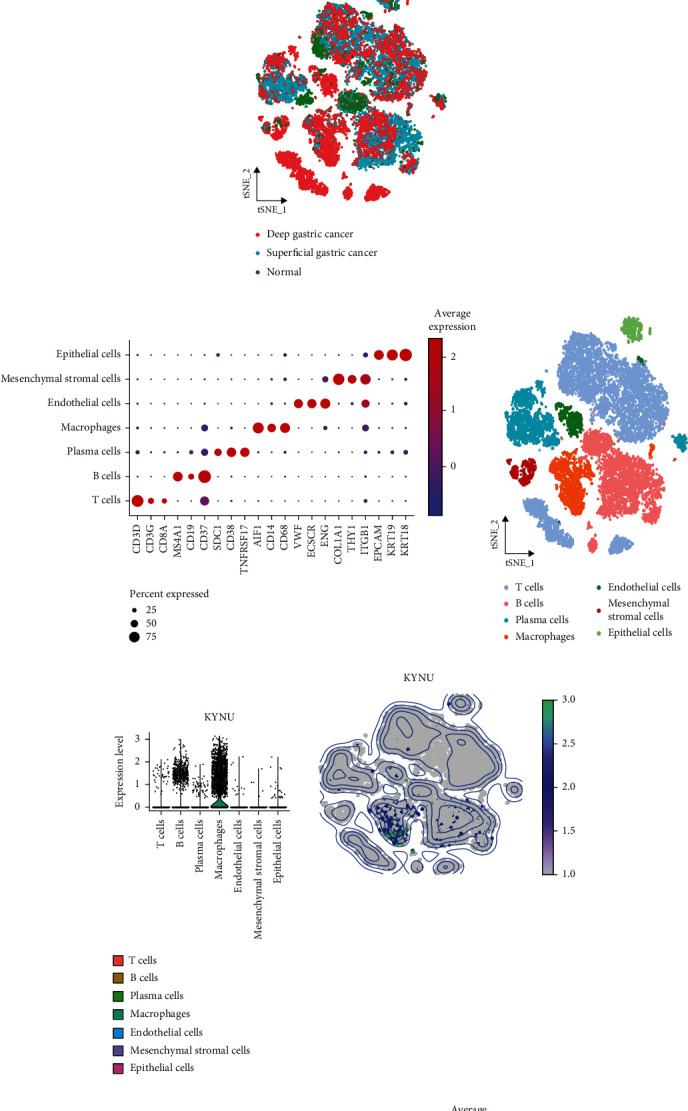
Overview of scRNA-seq data derived from ten GC and four normal gastric tissue samples. t-SNE plots categorized by (a) clusters, (b) sample origin, and (c) tissue type. (d) Specific marker genes corresponding to each cell subpopulation. (e) Annotated t-SNE plot. (f) Distribution of KYNU in different cell types. (g) Expression of KYNU in different tissues.

**Figure 7:**
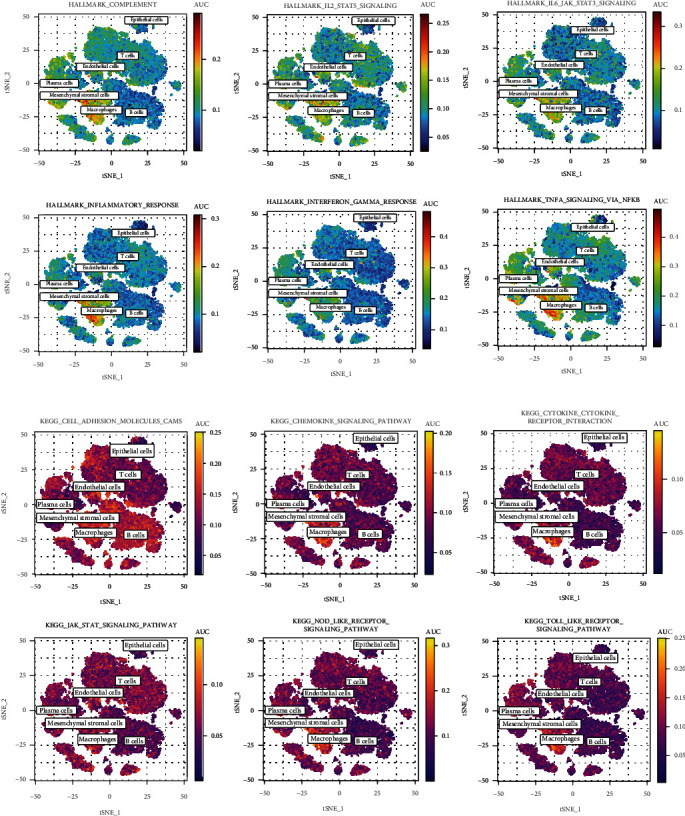
Signaling pathway activity in cellular subpopulations. (a) Pathway activity in the HALLMARK gene set. (b) Pathway activity in the KEGG gene set.

**Figure 8:**
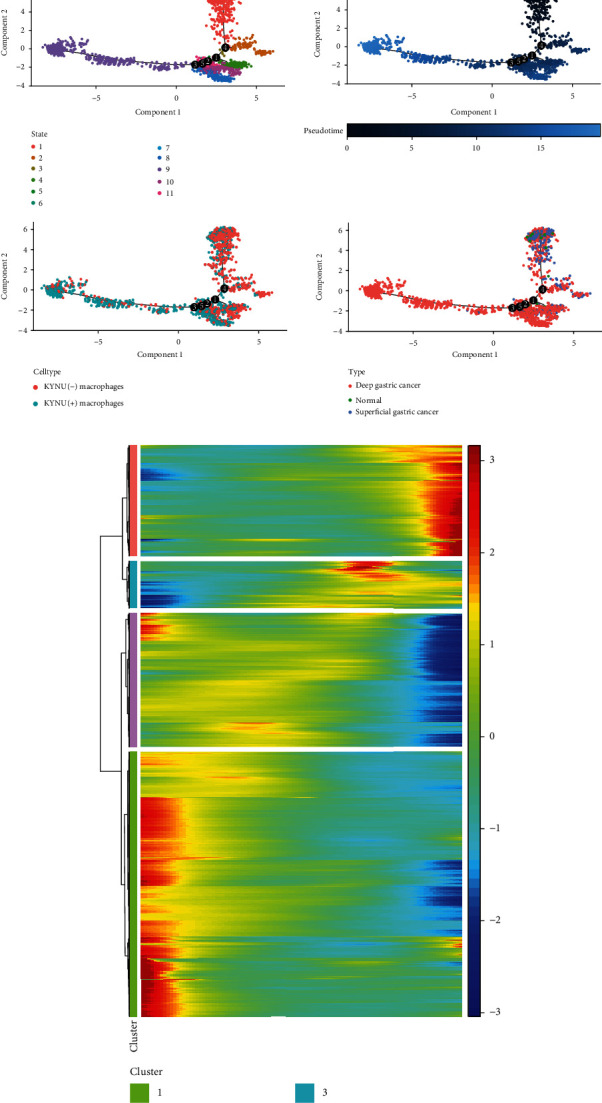
Pseudotime trajectory analysis of macrophage subpopulations. (a) Macrophages were divided into the KYNU(+) and KYNU(-) macrophage subpopulations. (b) The 10 most significantly upregulated differential genes in KYNU(+) and KYNU(-) macrophages. (c) Trajectories of the macrophage subpopulations plotted according to state, cell type, pseudotime, and tissue. (d) Dynamic gene expression in the pseudotime trajectory (red indicates upregulated gene expression and blue indicates downregulated gene expression). (e) Enrichment analysis of gene cluster 2.

From the scRNA-seq of ten GC and four paracancerous samples, we obtained 19,409 high-quality cells after quality control. These cells were clustered into 20 subpopulations (Figure 6(a)). The cell subpopulations were visualized by their sample origin (Figure 6(b)) and tissue type (Figure 6(c)) and were annotated according to specific marker genes (Figure 6(d)) as T, B, plasma, endothelial, mesenchymal stromal, and epithelial cells and macrophages (Figure 6(e)). We found that KYNU was mainly expressed on macrophages (Figure 6(f)) and in deep GC tissues, but not in normal gastric tissues (Figure 6(g)). We demonstrated that the signaling pathway activity from previous GSEA occurs in these cell subpopulations, and the signaling pathways involved in KYNU upregulation were highly active in macrophage subpopulations (Figures 7(a) and 7(b)). Subsequently, we isolated the macrophage subpopulation in GC and normal gastric tissues, which included 1943 macrophages. The PercentageFeatureSet function identified 1187 KYNU-expressing KYNU(+) macrophages and 756 non-KYNU-expressing KYNU(-) macrophages (Figure 8(a)). The FindMarkers function identified DEGs between the subpopulations of KYNU(+) macrophages and KYNU(-) macrophages, and Figure 8(b) depicts the top 10 most significantly upregulated genes in KYNU(+) macrophages, which were KYNU, SOD2, EREG, TIMP1, G0S2, IL1B, PLAUR, CXCL3, CCL20, and IL6. The GEPIA database confirmed that the other nine genes were significantly positively correlated with KYNU (*P* < 0.05) (Supplementary Figure. S1).

### 3.6. Construction of the Trajectories of Macrophages

To explore the developmental trajectory of KYNU-related macrophages, the Monocle2 algorithm was applied to simulate the trajectory of the macrophages, and Figure 8(c) shows that the macrophages were divided into 11 differentiation states. Based on the pseudotime trajectory, KYNU(+) macrophages were enriched more towards the end of the differentiation trajectory compared to KYNU(-) macrophages. Macrophages derived from normal tissues and superficial GC were distributed at the start of the trajectory, whereas those derived from deep GC were distributed across the middle and end sections. The genes expressed along the macrophage differentiation trajectory were grouped further into four clusters, and a heatmap was plotted (Figure 8(d)). KYNU was located in cluster 2, which included 528 genes (Supplementary Table S2). Additionally, the other nine most significantly upregulated differential genes in the KYNU(+) macrophage subpopulation were also located in cluster 2. Expressions of the genes in cluster 2 gradually increased along the trajectory, suggesting that these genes may promote the progression of GC together with KYNU at the single-cell level. Finally, we further conducted enrichment analysis on the genes in cluster 2 and found that TNFA signaling via NFkB and cytokine-cytokine receptor interaction pathways were the most significantly enriched (Figure 8(e)), indicating that these two pathways might be crucial for KYNU(+) macrophages to exert their procancer effects in GC.

### 3.7. Cell–Cell Communication Analysis

**Figure 9:**
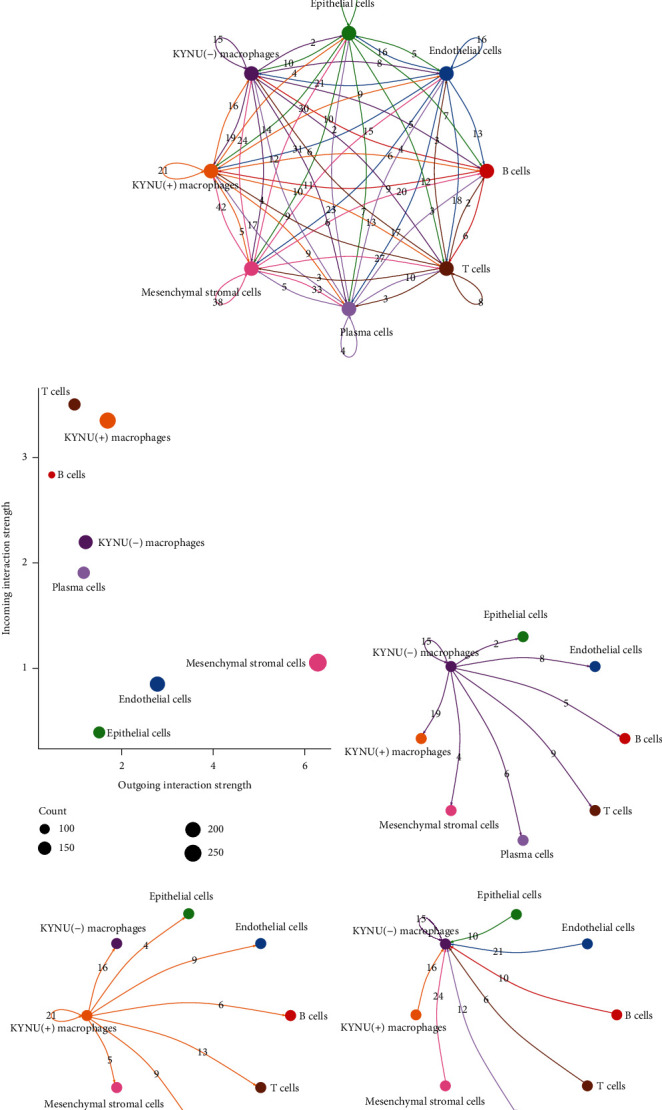
Role of KYNU-related macrophages in cell-cell communication. (a) GC-derived cell subpopulations were isolated, and the macrophage subpopulations were reannotated. (b) Exploration of cell interactions based on the CellPhoneDB algorithm, with larger numbers on the scale indicating stronger interactions. (c) Exploration of cell interactions based on the CellChat algorithm, with numbers representing the number of pathways involved in cell-cell interactions. (d) Strength and number of incoming and outgoing signals among cell subpopulations. Interactions of KYNU(-) and KYNU(+) macrophages with other cell subpopulations when acting as (e, f) signal senders and (g, h) signal receivers.

We first extracted the cell subpopulations from GC samples and reannotated the cell types (Figure 9(a)). We then applied the CellPhoneDB software and CellChat package to perform cell-cell interaction analysis to identify differences in the interactions between KYNU(+) macrophages and KYNU(-) macrophages with other cells in the GC microenvironment. The analysis from CellPhoneDB showed that, compared to KYNU(-) macrophages, KYNU(+) macrophages communicated with adjacent cells in the GC microenvironment more frequently (Figure 9(b)). We then performed further analysis using the CellChat algorithm (Figure 9(c)) and found that KYNU(+) macrophages had higher signal input and output intensities (Figure 9(d)). Moreover, KYNU(+) macrophages had more abundant signaling pathways than KYNU(-) macrophages, both as a signal sender and receiver (Figures 9(e)–9(h)). Supplementary Figure. S2 shows that KYNU(-) macrophages had a similar cell-to-cell signaling pattern to that of KYNU(+) macrophages. When macrophages acted as signal senders, the THBS (THBS1-CD47, THBS1-SDC1, and THBS1-SDC4) pathway was enriched in the cell interactions of the KYNU(+) macrophage subpopulation. When macrophages acted as signal receivers, pathways such as TENASCIN (TNXB-SDC4), THY1 (THY1-(ITGAX+ITGB2)), GALECTIN (LGALS9-HAVCR2), ICAM (ICAM1-(ITGAX+ITGB2)), ANNEXIN (ANXA1-FPR1), and COMPLEMENT (C3-(ITGAX+ITGB2)) were enriched in the KYNU(+) macrophage subpopulation. Notably, compared to KYNU(-) macrophages, the KYNU(+) macrophage subpopulation overexpressed the SDC4 receptor and had stronger cell-cell interactions with mesenchymal stromal cells. Our results suggest that KYNU may exert its carcinogenic effects at the cellular level through the aforementioned ligand-receptor pairs.

### 3.8. Transcriptional Regulatory Network of KYNU(+) Macrophages

**Figure 10:**
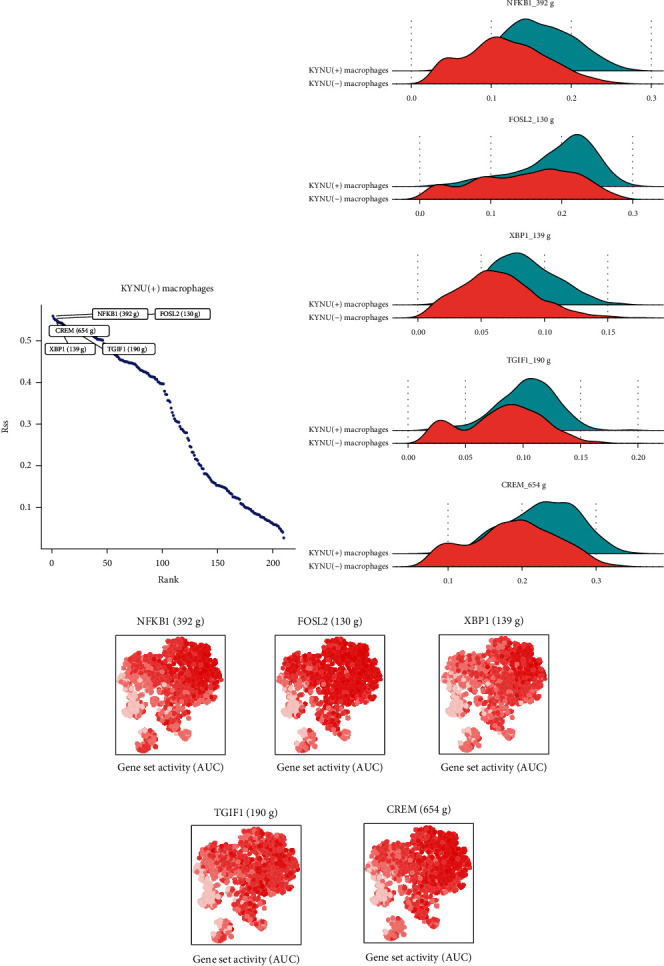
Transcriptional regulation of KYNU in GC. (a) Top five TFs with the highest specificity among KYNU(+) macrophages. (b) Ridge plot showing the expression of the top five TFs in KYNU(-) and KYNU(+) macrophages. (c) t-SNE plot showing the activity levels of the top five TFs in the macrophage subpopulations.

The SCENIC algorithm was applied to analyze the specific TFs in KYNU(+) macrophages. RSS, NFKB1, FOSL2, XBP1, TGIF1, and CREM were identified as the five TFs with the highest specificity in the KYNU(+) macrophage subpopulation (Figure 10(a)). Furthermore, Figures 10(b) and 10(c) show that these five TFs were more active in the KYNU(+) macrophage subpopulation than in the other macrophage subpopulations. Therefore, we speculate that KYNU(+) macrophages are subjected to the upregulation of these TFs, thereby promoting the formation of an adverse TME.

### 3.9. Relationship between KYNU(+) Macrophages and Tumor Phenotype and Survival Rate

**Figure 11:**
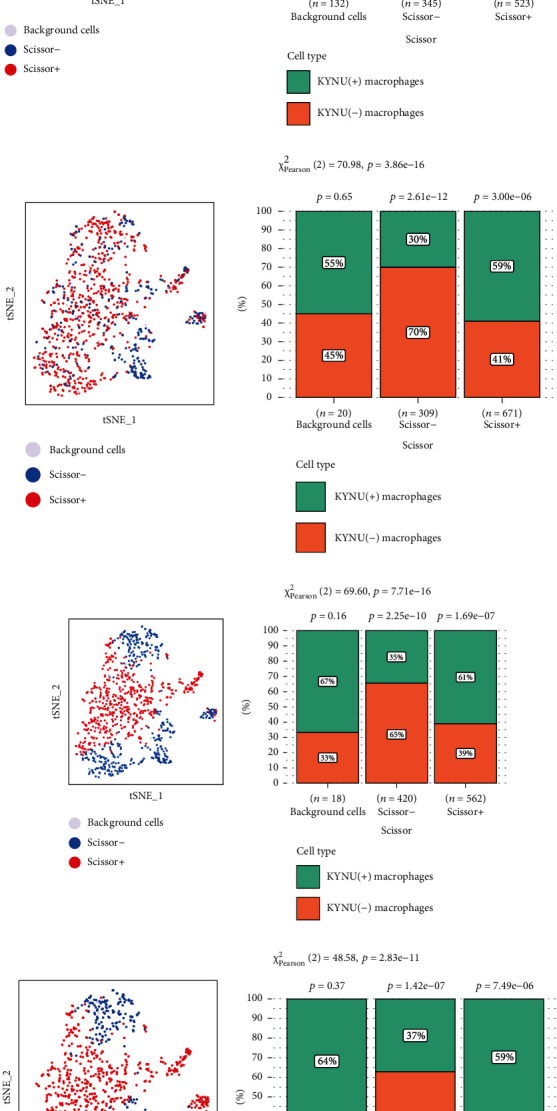
Scissor identification of KYNU-related macrophages. (a) 1000 macrophages were included in the Scissor analysis. Relationship between (b) tumor vs. normal, (c) survival status, (d) N stage, (e) TNM stage, and (f) grade and KYNU(+) and KYNU(-) macrophages.

During analysis of the clinical features, we found that KYNU was significantly correlated with grade, N stage, and TNM stage. Therefore, the phenotype indicator values such as “Tumor,” “Dead,” “N1-3,” “StageIII-IV,” and “Grade 3” were set as “1,” while those of “Normal,” “Alive,” “N0,” “StageI-II,” and “Grade 1–2” were set as “0”, and 500 KYNU(+) macrophages and 500 KYNU(-) macrophages were selected (Figure 11(a)). The Scissor algorithm was used to calculate the number of Scissor+ cells, Scissor- cells, and background cells for each clinical feature. Compared to KYNU(-) macrophages, KYNU(+) macrophages accounted for a higher proportion of Scissor+ cells with the “Tumor,” “Dead,” “N1-3,” and “StageIII-IV” phenotypes (*P* < 0.05); whereas for Scissor- cells with the “Normal,” “Alive,” “N0,” and “StageI-II” phenotypes, KYNU(-) macrophages accounted for a higher proportion (*P* < 0.05) than KYNU(+) (Figures 11(b)–11(e)). Notably, there was no significant difference in the correlation between KYNU(+) and KYNU(-) macrophages and grade (Figure 11(f)) (*P* > 0.05). Our findings demonstrated that KYNU(+) macrophages were positively correlated with adverse clinical phenotypes in patients with GC.

### 3.10. Knockdown of KYNU Inhibits Gastric Adenocarcinoma Cell Growth and Metastasis In Vitro

**Figure 12:**
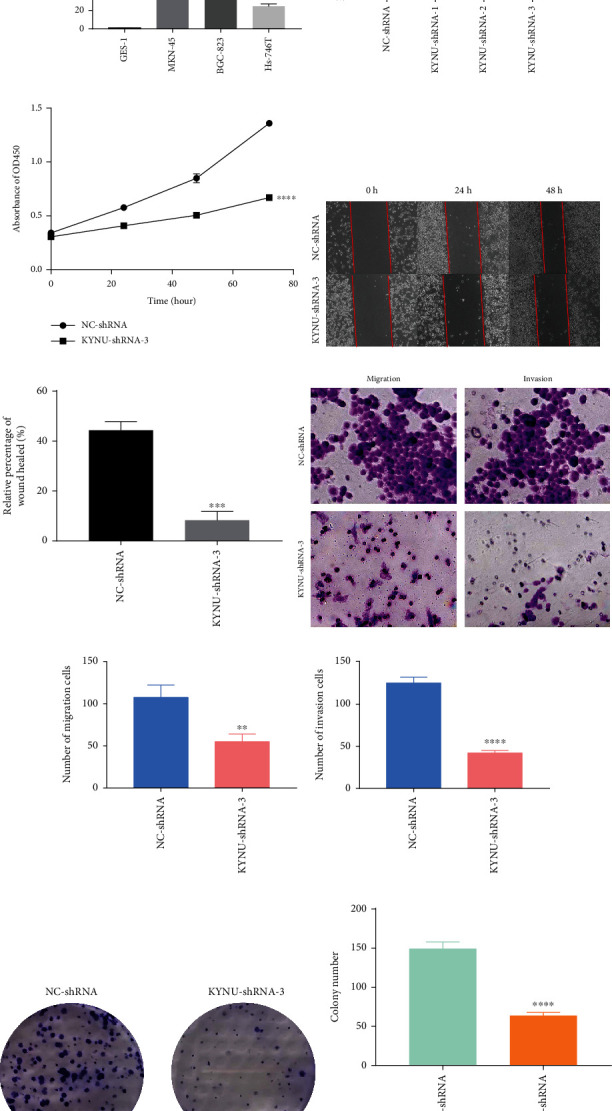
KYNU expression in GC and the effect of its deletion on GC cell proliferation and invasion (a) qRT-PCR analysis of KYNU differential expression in normal gastric and GC cells. (b) In BGC-823 cells, the downregulation of KYNU-shRNA-3 was most significant. (c) CCK8 assay shows the proliferation and viability of BGC-823 cells after KYNU knockout. (d, e) Wound healing assay shows the migration ability of BGC-823 cells after KYNU knockout. (f, g) Transwell assay was used to analyze the difference in the invasion and migration abilities of BGC-823 cells between the NC-shRNA and KYNU-shRNA groups. (h, i) Colony formation assays were performed to compare the number of BGC-823 clones between the NC-shRNA and KYNU-shRNA groups (^∗^*P* < 0.05, ^∗∗^*P* < 0.01, ^∗∗∗^*P* < 0.001, and ^∗∗∗∗^*P* < 0.000).

In vitro cellular experiments were performed to explore the effects of KYNU on the proliferation and migration of GC cells. qRT-PCR analysis showed that KYNU expression was significantly upregulated in GC cells (MKN-45, BGC-823, and Hs-746T) compared with normal gastric cells (GES-1), and KYNU was most significantly expressed in BGC-823 cells (Figure 12(a)). Thus, BGC-823 cells were selected for subsequent functional experimental analyses. Three types of shRNA were used to suppress KYNU expression in BGC-823 cells. The qRT-PCR assay revealed that KYNU-shRNA-3 had the best silencing efficiency (Figure 12(b)). KYNU-shRNA-3 was used in subsequent experiments. The results of the CCK8 assay showed that, following KYNU gene knockout, the number of BGC-823 cells was significantly reduced compared to that in the NC-shRNA group (Figure 12(c)). The results of the wound healing assay indicated that after KYNU knockout the GC cells exhibited an attenuated healing ability across the different time periods (Figures 12(d) and 12(e)). The results of the transwell assay showed that the migration and invasion abilities of BGC-823 cells were significantly reduced after KYNU gene knockout (Figures 12(f) and 12(g)). The results of the colony formation assay showed that KYNU deletion significantly reduced the number of new BGC-823 clones (Figures 12(h) and 12(i)).

## 4. Discussion

Tryptophan metabolism not only serves as an important pathway for tumors to escape immune surveillance in an immune cell infiltration environment but also as a critical bridge between inflammation and cancer [27]. As a key enzyme in the tryptophan metabolic pathway, KYNU overexpression promotes the accumulation of 3-hydroxyanthranilic acid, effectively reducing the sensitivity of cancer cells to ferroptosis [28]. Al-Mansoob et al. demonstrated that the interaction between CD44 and hyaluronan promotes breast cancer cell invasion by activating downstream KYNU expression [29]. In addition, TDO2 promotes poor prognosis in colon cancer, and Zhao et al. found that knocking out TDO2 in colon cancer cells inhibited the invasion and migration of cancer cells and downregulated KYNU expression [30]. Furthermore, Li et al. identified KYNU as a potential biomarker for chemotherapy sensitivity in laryngeal squamous cell carcinoma [31].

Based on bulk RNA-seq, scRNA-seq, and in vitro cell experiments, we have demonstrated, for the first time, that KYNU is overexpressed in GC and is associated with poor prognosis, as well as being an independent risk factor. The KYNU-nomogram constructed after integrating clinical features has excellent predictive value.

KYNU expression is significantly upregulated in atopic dermatitis and psoriasis, positively correlates with inflammation severity, and is significantly downregulated after treatment [26]. This finding was confirmed in our enrichment analysis, where we found that in GC, upregulated KYNU expression activated pathways such as inflammatory response, COMPLEMENT, IL2/STAT5 signaling, IL2/STAT5 signaling, and TNFA signaling via NFkB. Moreover, KYNU played a significant role in the chemokine signaling pathway, NOD-like receptor signaling pathway, and Toll-like receptor signaling pathway, suggesting that KYNU upregulation is involved in GC progression via multiple pathways.

ssGSEA showed that infiltration of CD8+ T cells and Tregs increased in the high KYNU GC group. Liu et al. found that IL-2 promotes the proliferation and activation of CD8 T cells during early-stage tumor development and induces the failure of CD8 T cells through the IL-2/STAT5 pathway during late-stage development [32]. The combined results of the enrichment analysis demonstrated that KYNU upregulation is involved in the activation of the IL-2/STAT5 pathway, which may be related to the increased infiltration of CD8 T cells in the high KYNU expression GC group. Enarsson et al. reported that increased numbers of Tregs in gastric tissue suppress anti-infection and antitumor immune responses, promoting GC progression [33].

Additionally, the infiltration of mast cells, macrophages, neutrophils, and plasmacytoid dendritic cells (pDCs) in the GC microenvironment is closely related to the high expression of KYNU. CXCL12-CXCR4 chemotaxis-mediated mast cell infiltration promoted the suppression of cellular immunity in the GC microenvironment by upregulating PD-L1 expression [34]. Neutrophil infiltration increases the risk of postoperative infection and metastasis in GC through TGF-*β* signaling [35]. In addition, the increase in macrophage infiltration in GC not only weakens the therapeutic responsiveness to 5-fluorouracil but also synergistically promotes the formation of an immunosuppressive microenvironment with Treg infiltration [36]. Furthermore, pDCs induce poor prognosis in tumors through the amplification of Tregs [37]. Similarly, functional pathways such as CCR, parainflammation, type 1 IFN and type 2 IFN responses, antigen-presenting cell (APC) coinhibition, and T cell coinhibition were active in the KYNU high-expression group. Mollica Poeta et al. reported that the CCR-related pathway participated in tumor cell metastasis and had the potential to serve as a target for antitumor treatment [38]. Parainflammation is common in various cancers and is associated with tumor progression [39]. Type 1 IFN has been shown to promote immune suppression and the progression of gastric diseases [40]. In addition, the activation of APC coinhibition and T cell coinhibition increases the risk of poor prognosis [41]. In this study, immune checkpoint analysis revealed the upregulated expression of immune checkpoints such as HAVCR2, PDCD1 (PDCD1), LAG3, CTLA4, and CD274 (PD-L1) in the KYNU high-expression group. These findings suggested that the upregulation of KYNU might promote GC progression by enhancing the infiltration of harmful immune cells in the TME and inducing the formation of an immunosuppressive state in the GC microenvironment.

We analyzed the biological characteristics of KYNU at the single-cell level in GC and found that macrophages expressed the highest levels of KYNU. Harden et al. found that KYNU was expressed in CD163+ macrophages in psoriasis [26], consistent with our study finding. KYNU was mainly expressed in cell subpopulations derived from deep GC samples. The differential analysis identified the 10 most significantly upregulated DEGs in KYNU(+) macrophages, and the GEPIA online tool confirmed that the nine other DEGs than KYNU were significantly positively correlated with KYNU. These genes may promote GC progression along with KYNU.

Cell trajectory analysis showed that KYNU(+) macrophages were distributed in the middle and end of the differentiation trajectory, consistent with the direction of differentiation of macrophages from superficial GC to deep GC. Additionally, the expression of KYNU and its nine coexpressed genes gradually increased along the differentiation trajectory. The enrichment analysis of genes consistent with the KYNU expression pattern (cluster 2) revealed that TNFA signaling via the NF-*κ*B and cytokine-receptor interaction pathways were the most significantly enriched, consistent with previous GSEA results. Ju et al. found that tumor-associated macrophages in the GC microenvironment released TNF-*α* and IL6, inducing the upregulation of PD-L1 and promoting immune escape, a mechanism closely related to the activation of the NF-*κ*B pathway [42]. Omar et al. discovered through in vitro experiments that the NF-*κ*B pathway induced TIMP1 overexpression and enhanced the proliferation potential of GC cells [43]. Moreover, the upregulated expression of SOD2, IL1B, PLAUR, CXCL3, and CCL20 promoting cancer cell invasion in other tumors depended on the NF-*κ*B mechanism [44–48]. Our results suggest that KYNU and its coexpressed genes may promote GC development via TNFA signaling through the NFKB pathway.

Cell communication analysis can effectively reveal the input and output modes of ligand-receptor signals between different cell subpopulations [20]. This study investigated the differences in cell communication between KYNU(+) macrophages and KYNU(-) macrophages. The results revealed that many unique pathways are enriched in the communications involving the KYNU(+) macrophage subpopulation, such as THBS (THBS1-CD47, THBS1-SDC1, THBS1-SDC4), TENASCIN (TNXB-SDC4), THY1 (THY1-(ITGAX+ITGB2)), GALECTIN (LGALS9-HAVCR2), ICAM (ICAM1-(ITGAX+ITGB2)), ANNEXIN (ANXA1-FPR1), and COMPLEMENT (C3-(ITGAX+ITGB2)). In previous research, THBS and CD47 were found to be jointly involved in inhibiting antitumor immunity [49]. The interaction between THBS1 and SDC1 expressed in malignant gliomas promotes tumor cell invasion [50]. Outeiro-Bernstein et al. discovered that the binding of THBS1 to SDC4 inhibited the apoptosis of vascular endothelial cells and maintained their adhesion function [51]. Furthermore, TNXB is significantly expressed in malignant mesothelioma [52]. SDC4 is upregulated in various tumors, and Yang et al. reported that SDC4 promotes liver cancer progression, possibly related to MAPK, focal adhesion, and angiogenesis signaling [53]. Moreover, in thyroid cancer and lung adenocarcinoma, the SDC4-mediated epithelial-mesenchymal transformation process was found to be closely related to the invasion and migration of tumor cells [54]. SDC4 was also found to be overexpressed in ovarian cancer and fibrosarcoma [55, 56]. THY1 is overexpressed in GC and inhibited GC cell apoptosis by upregulating the SPARC protein [57]. The LGALS9−HAVCR2 ligand-receptor pair is an important target for mediating immune escape in a wide range of cancers such as leukemia, breast cancer, and melanoma [58, 59]. Ni et al. confirmed through in vitro experiments that knocking out ITGAX could significantly inhibit the proliferation and migration of melanoma cells [60]. Meanwhile, ITGB2 is involved in the progression of oral squamous cell carcinoma via the PI3K-AKT-mTOR pathway [61]. In addition, Chen et al. proposed ICAM1 as a new marker for poor prognosis in GC [62]. Here, we found that the KYNU(+) macrophage subpopulations participated in the communication via the COMPLEMENT signaling pathway, which aligns with the results we obtained in the enrichment analysis. However, the biological mechanisms of the interactions between TNXB-SDC4, THY1-(ITGAX+ITGB2), ICAM1-(ITGAX+ITGB2), and C3-(ITGAX+ITGB2) ligand-receptor pairs in cancer remain unclear and warrant further investigation. Furthermore, KYNU(+) macrophages demonstrated significant expression of the SDC4 receptor and communicate and interact with mesenchymal stromal cells. As mentioned earlier, the upregulation of SDC4 promotes poor progression in various tumors. Thus, we hypothesize that KYNU(+) macrophages can cooperate with mesenchymal stromal cells in the TME to exacerbate GC progression.

The dysregulation of TFs greatly increases the risk of tumor progression. Therefore, drugs targeting TFs have been widely employed in cancer therapy [63]. In this study, using the SCENIC algorithm, we identified NFKB1, FOSL2, XBP1, TGIF1, and CREM as specifically expressed transcription factors in the KYNU(+) macrophage subpopulations. NFKB1 is involved in the regulation of tumor-associated macrophage polarization in colorectal cancer [64]. *β*-Catenin regulates the transition from M1 to M2 macrophages by activating FOSL2, leading to poor prognosis in patients with lung adenocarcinoma [65]. Activation of XBP1 in TAMs promotes the progression of colorectal cancer, which may be related to the upregulation of SIRP*α* and THBS1, and the inhibition of macrophage phagocytosis [66]. Similarly, Zhang et al. confirmed through in vitro experiments that knocking out TGIF1 significantly inhibited the proliferation and invasion of GC cells [67]. Moreover, Yu et al. found that high expression of CREM promoted the polarization of M2 macrophages and the progression of GAEC [68]. This offers new insights into the molecular mechanisms underlying KYNU's promotion of poor prognosis in GC. Combined with the clinical phenotypes in the TCGA-STAD cohort, using the Scissor algorithm, we found that Scissor+ cells associated with the “Tumor,” “Dead,” “N1-3,” and “StageIII-IV” phenotypes exhibited a higher distribution of KYNU(+) macrophages. Conversely, KYNU(-) macrophages were mainly distributed in Scissor- cells associated with the “Normal,” “Alive,” “N0,” and “StageI-II” phenotypes. This suggests that infiltration of the KYNU(+) macrophage subpopulation in the TME may exacerbate GC progression and poor prognosis.

Our study revealed that KYNU expression was significantly higher in GC than in normal gastric cells and was highest in the BGC-823 cell line. Furthermore, the inhibition of KYNU expression significantly impeded the proliferation, invasion, and migration of BGC-823 cells.

There are a few limitations to this study. The biological effects of KYNU were mainly explored based on publicly available data and in vitro cell experiments related to GC. Although this is the first study to elucidate the relationship between KYNU and macrophages in GC and reveal the role of KYNU(+) macrophages in the TME, the underlying mechanism through which KYNU(+) macrophages facilitate GC progression needs to be explored further.

## 5. Conclusion

We found that KYNU exerted tumor-promoting effects in GC and that its high expression was associated with malignant phenotypes which could lead to poor prognosis. Analysis of scRNA-seq data showed that, as a biomarker of GC-associated macrophages, KYNU could induce the formation of an unfavorable TME. In addition, the infiltration of KYNU(+) macrophages was consistent with the tendency of GC invasion, which usually expressed ligand-receptor pairs that promoted tumourigenesis and progression. KYNU(+) macrophages were regulated by prooncogenic transcription factors. In vitro experiments confirmed that KYNU deficiency inhibited GC progression. This study comprehensively decoded the role of KYNU in gastric cancer, identified potential targets for individualised treatment of GC, and provided a theoretical basis for the future design of drugs targeting KYNU and KYNU(+) macrophage-related drugs.

## Data Availability

The original contributions presented in the study are included in the article/Supplementary Material. Further inquiries can be directed to the corresponding author.

## Supplementary Materials

Supplementary 1 Supplementary Figure. S1: exploring the relationship between nine DEGs and KYNU based on the GEPIA database.

Supplementary 2 Supplementary Figure. S2: ligand-receptor pairs involved in KYNU(-) and KYNU(+) macrophage activity as (A, B) signal senders and (C, D) signal receivers.

Supplementary 3 Supplementary Table S1: KYNU expression and clinical characteristics in GC.

Supplementary 4 Supplementary Table S2: gene clusters expressed along the pseudotime trajectory.
