# Supplementary figures and images for: KYNU as a Biomarker of Tumor-Associated Macrophages and Correlates with Immunosuppressive Microenvironment and Poor Prognosis in Gastric Cancer
Source: Int J Genomics. 2023 Nov 2;2023:4662480. doi: 10.1155/2023/4662480 (PMC10635752; doi:10.1155/2023/4662480)

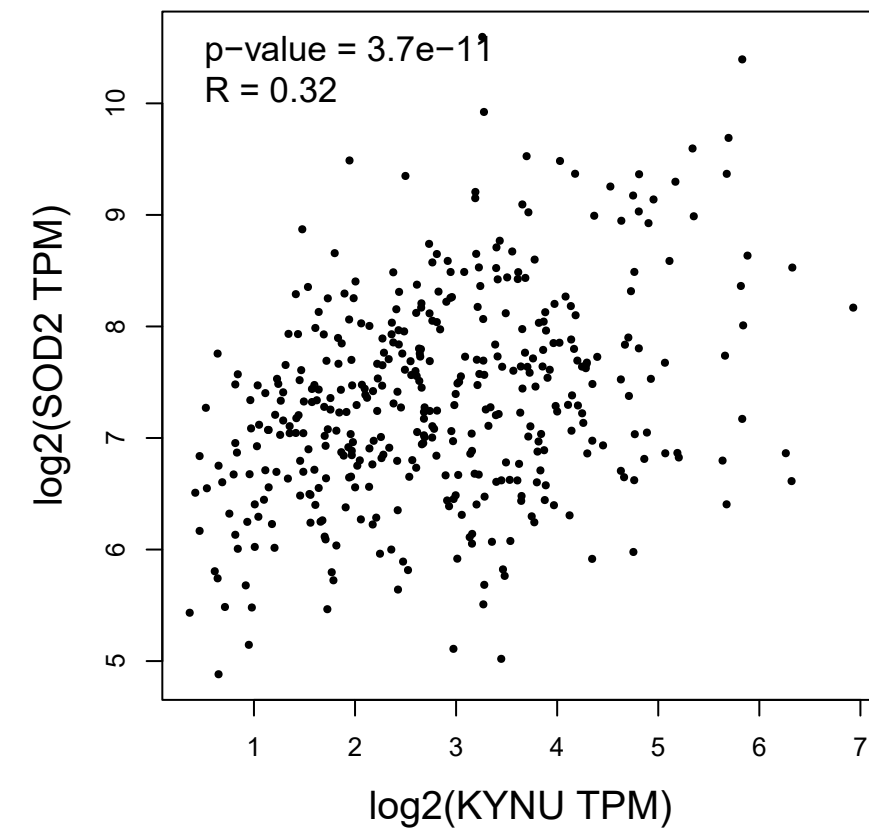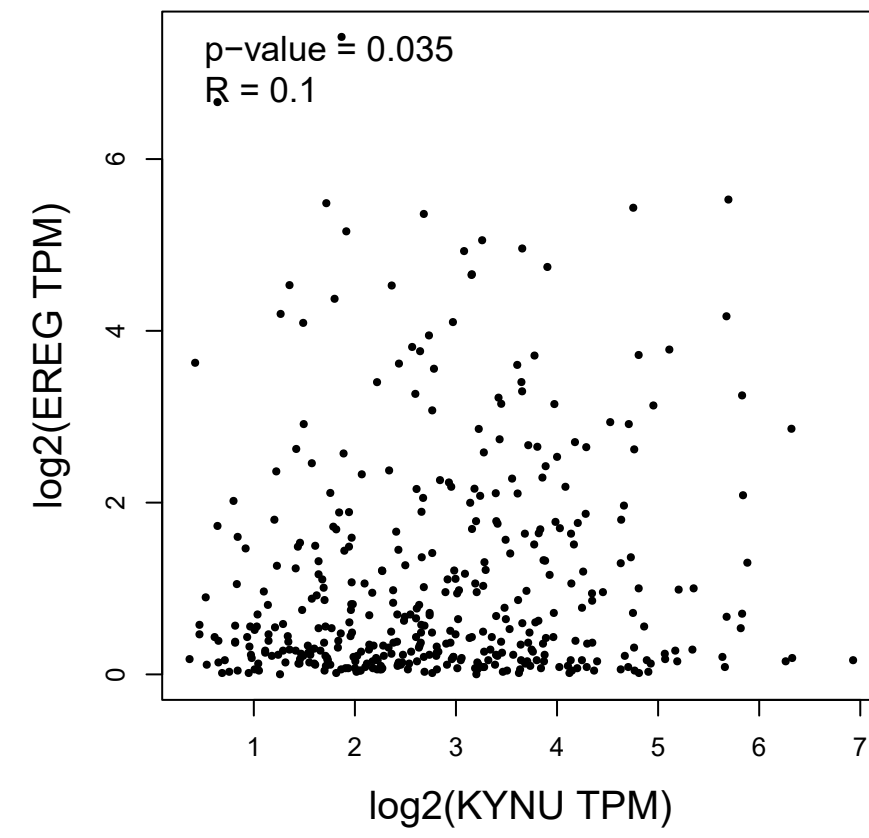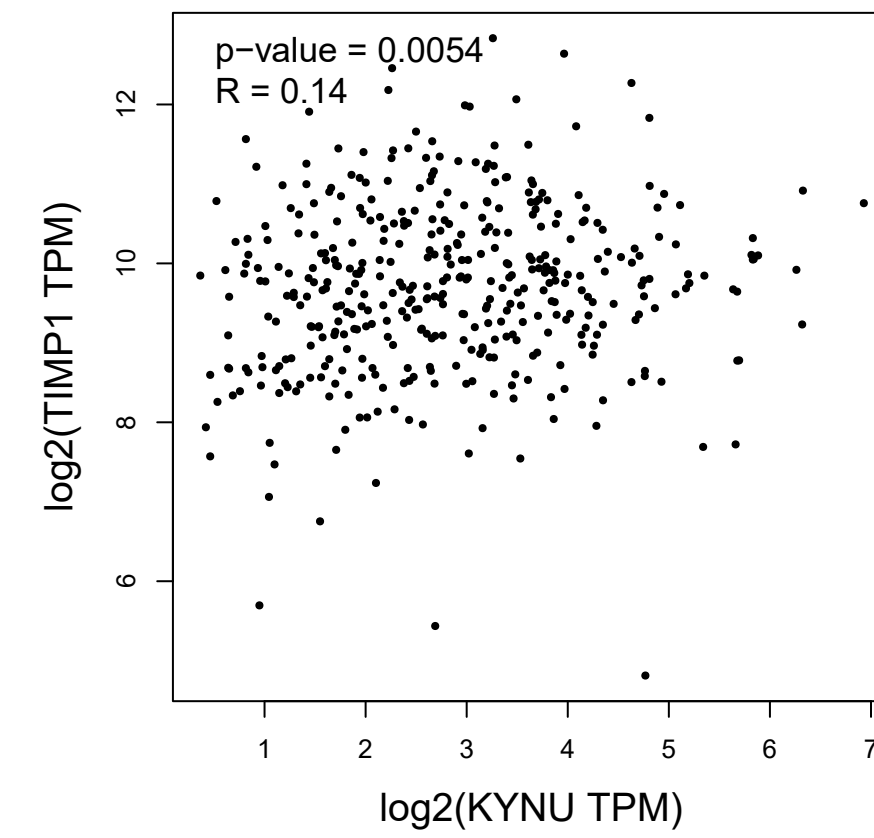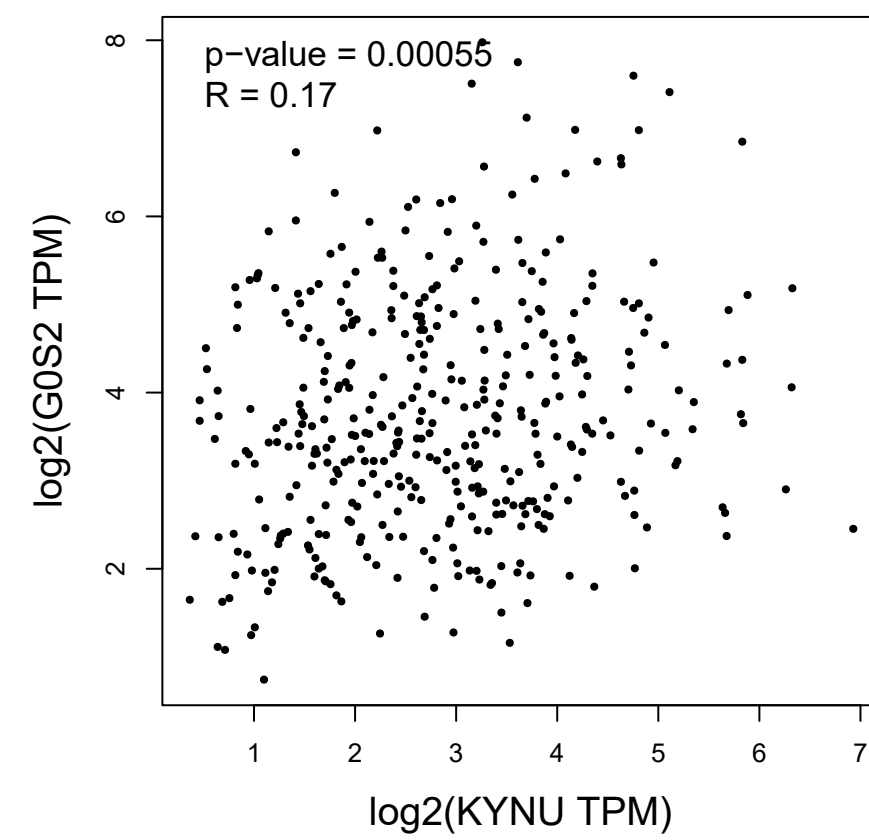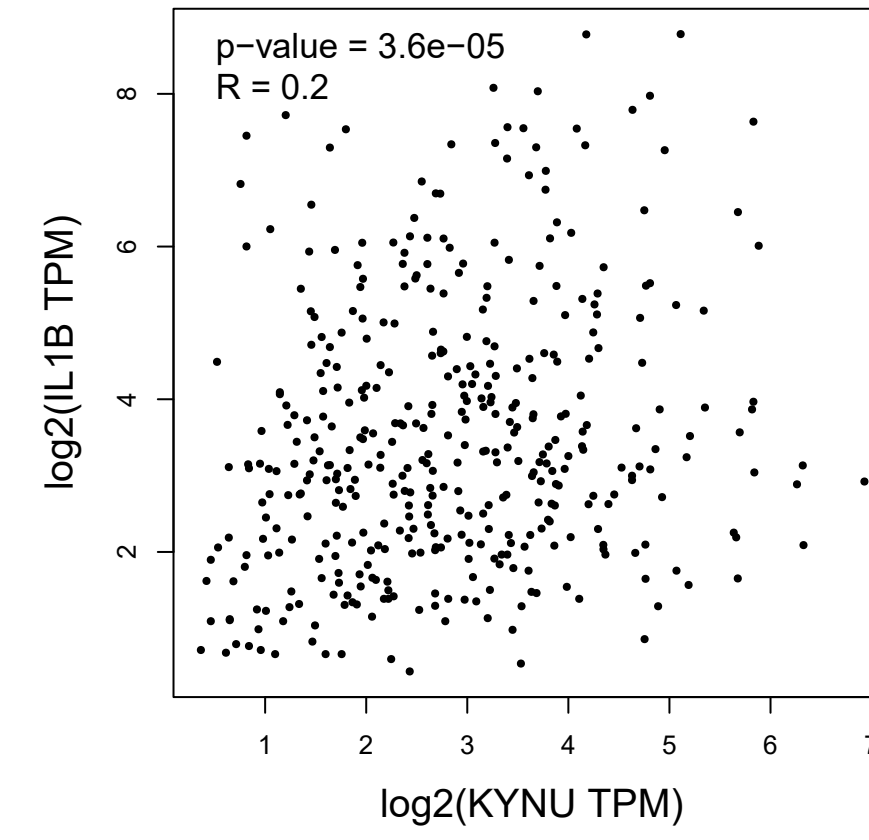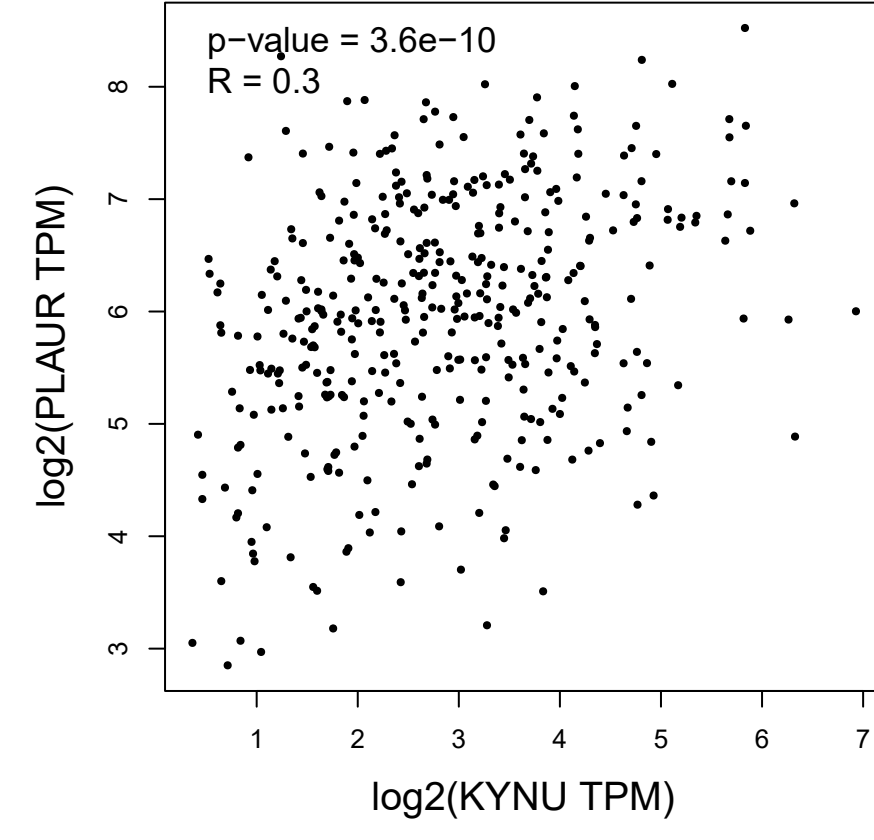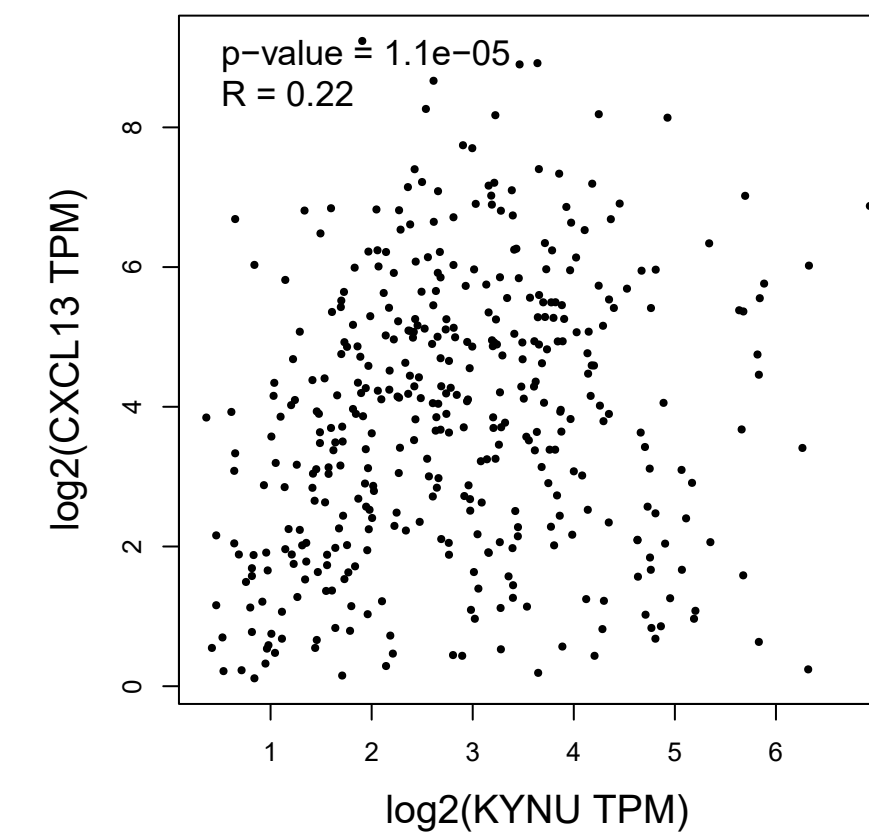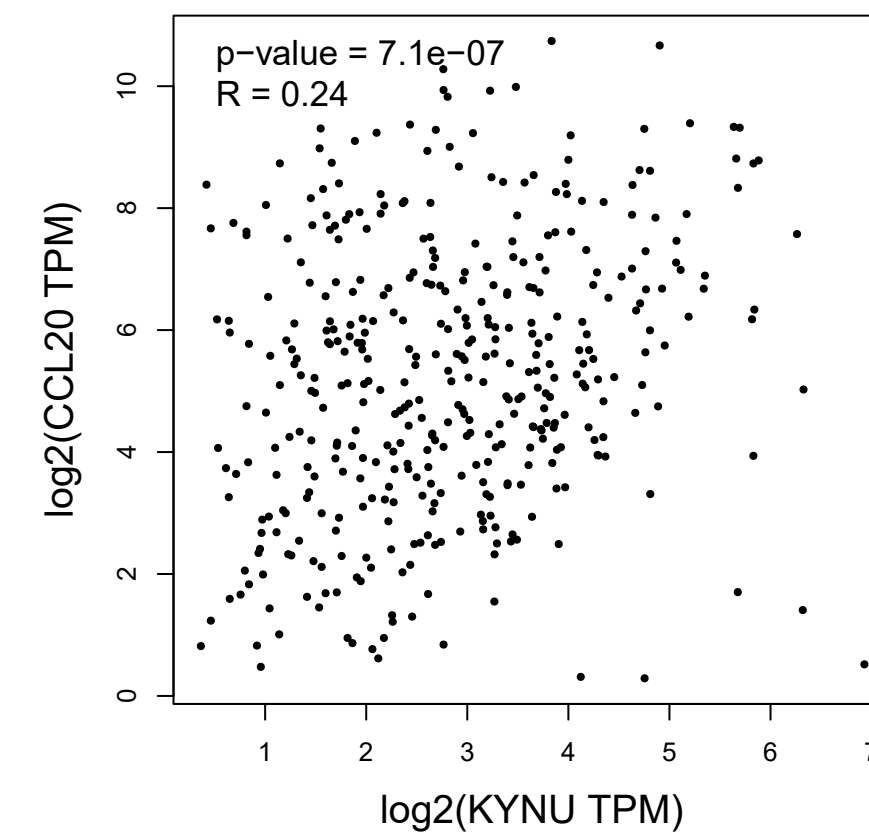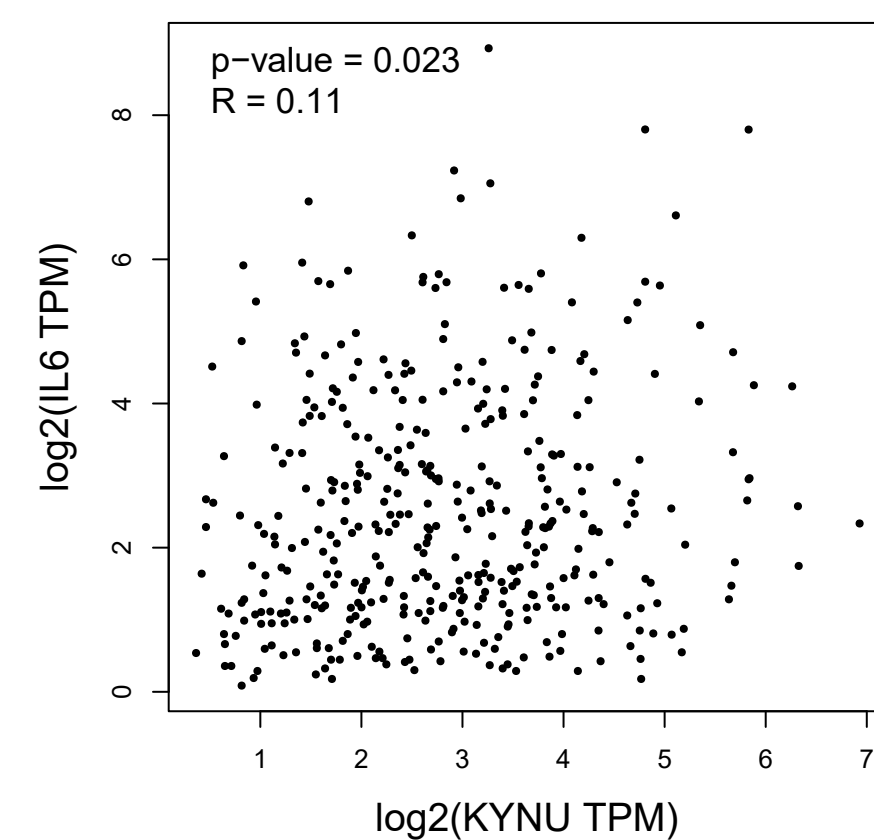

Supplement: Supplementary 1 — Supplementary Figure. S1: exploring the relationship between nine DEGs and KYNU based on the GEPIA database. [file 4662480.f1.pdf]

A

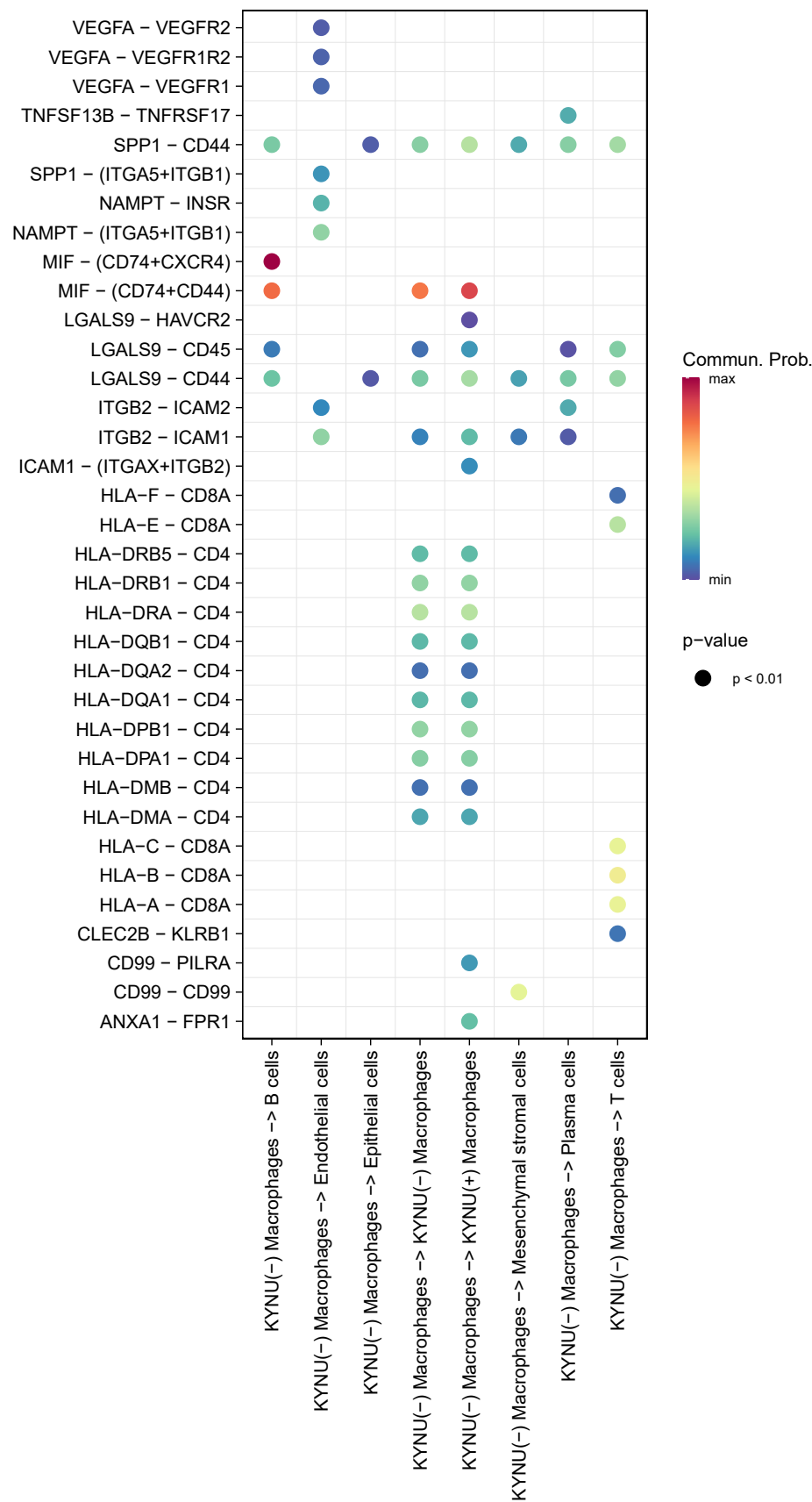

B

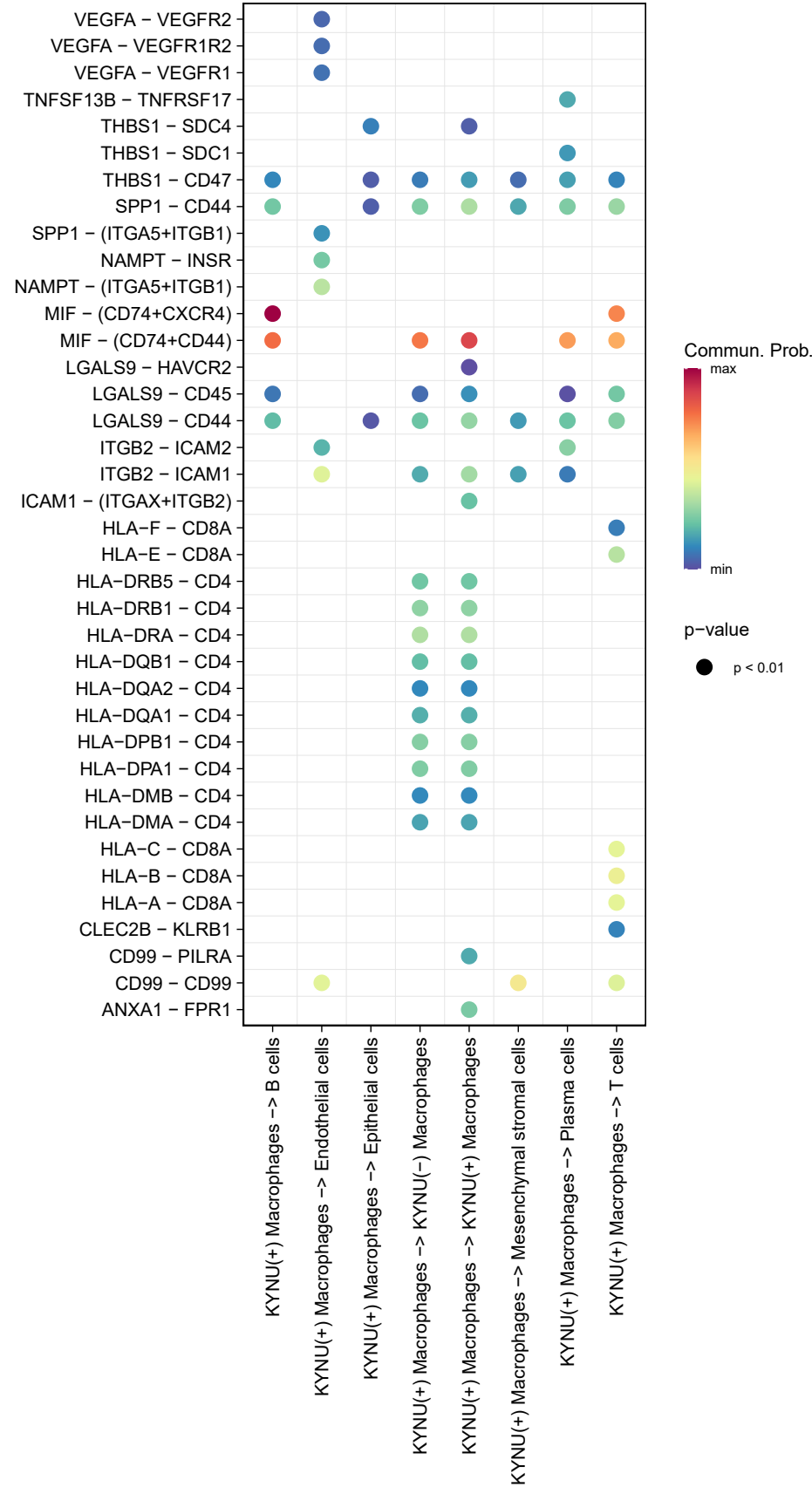

C

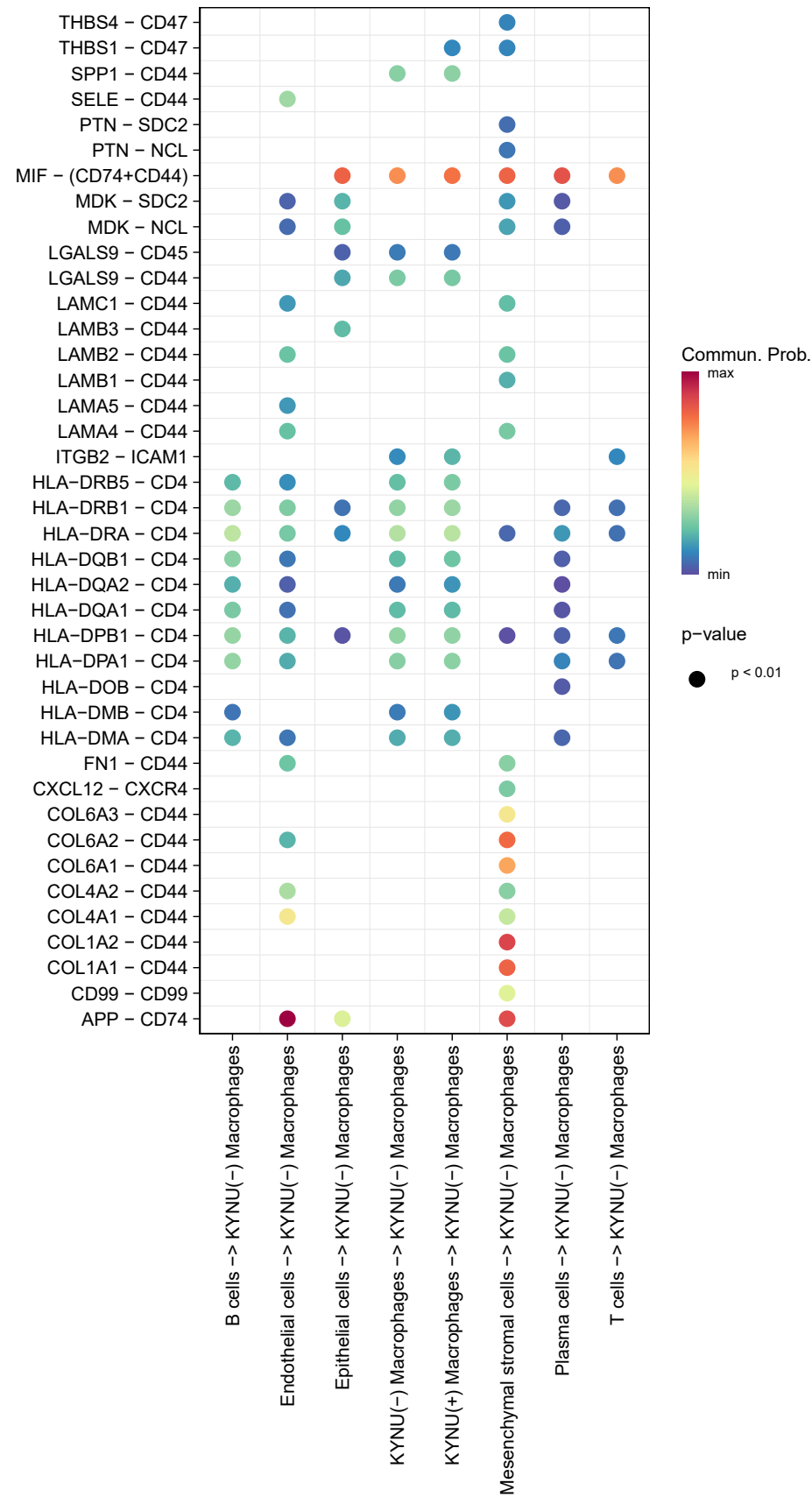

D

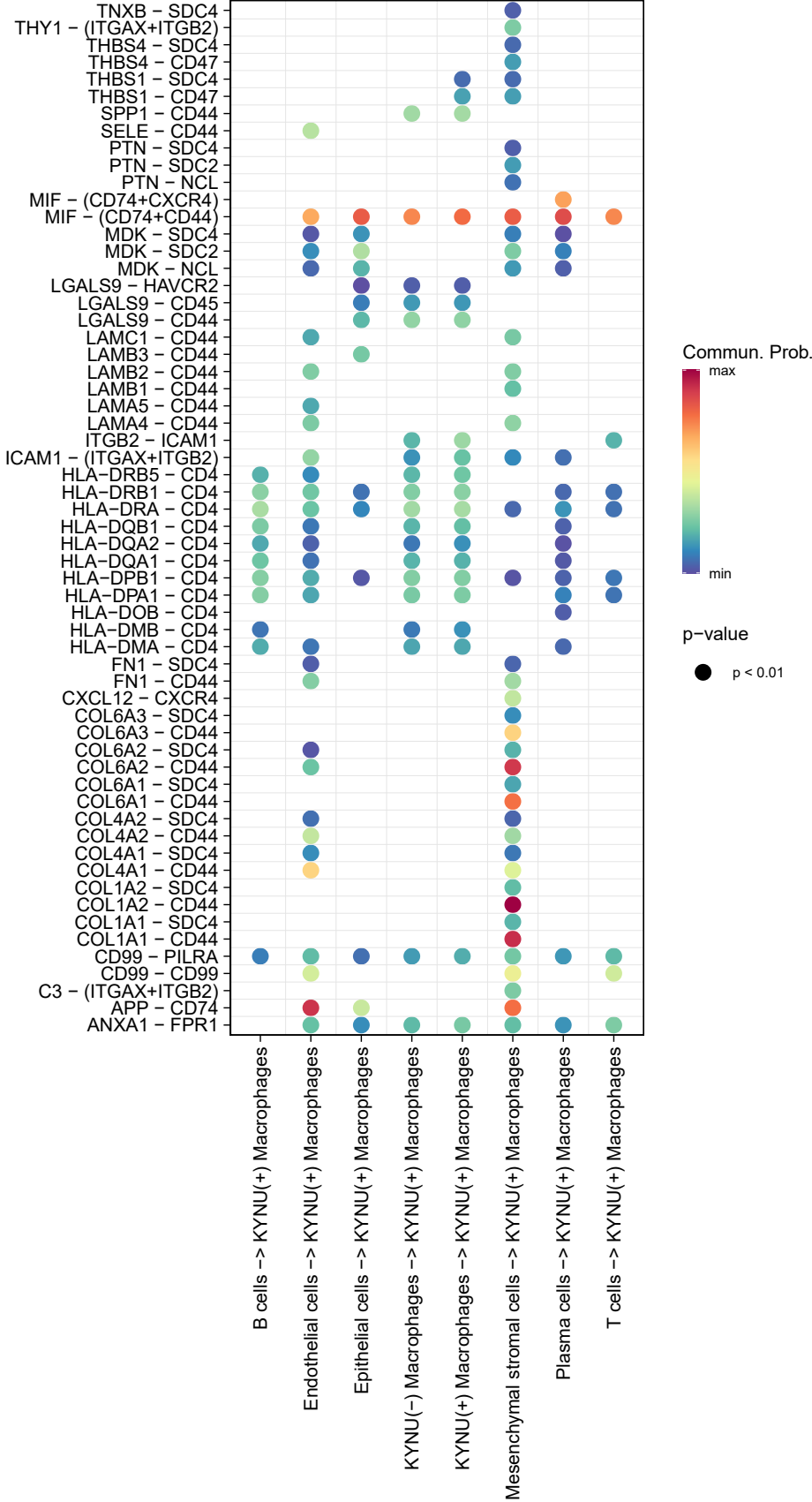

Supplement: Supplementary 2 — Supplementary Figure. S2: ligand-receptor pairs involved in KYNU(-) and KYNU(+) macrophage activity as (A, B) signal senders and (C, D) signal receivers. [file 4662480.f2.pdf]
