# Supplementary material for: KYNU as a Biomarker of Tumor-Associated Macrophages and Correlates with Immunosuppressive Microenvironment and Poor Prognosis in Gastric Cancer
Source: Int J Genomics. 2023 Nov 2;2023:4662480. doi: 10.1155/2023/4662480 (PMC10635752; doi:10.1155/2023/4662480)
Supplement: Supplementary 3 — Supplementary Table S1: KYNU expression and clinical characteristics in GC. [file 4662480.f3.pdf]

Supplementary Table S1: KYNU expression and clinical characteristics in GC.

Characteristic	Case(n)	KYNU low (n = 159)	KYNU high (n = 158)	P value
Age	≥65	94	89	0.615
	＜65	65	69	
Gender	Female	68	52	0.070
	Male	91	106	
Histologic Grade	G1	2	5	0.043
	G2	64	44	
	G3	93	109	
TNM Stage	Stage I	25	17	0.417
	Stage II	53	48	
	Stage III	66	73	
	Stage IV	15	20	
T stage	T1	9	6	0.884
	T2	31	32	
	T3	75	77	
	T4	44	43	
N stage	N0	57	42	0.1339
	N1	40	43	
	N2	36	33	
	N3	26	40	
M stage	M0	150	145	0.369
	M1	9	13	
