# Supplementary material for: KYNU as a Biomarker of Tumor-Associated Macrophages and Correlates with Immunosuppressive Microenvironment and Poor Prognosis in Gastric Cancer
Source: Int J Genomics. 2023 Nov 2;2023:4662480. doi: 10.1155/2023/4662480 (PMC10635752; doi:10.1155/2023/4662480)
Supplement: Supplementary 4 — Supplementary Table S2: gene clusters expressed along the pseudotime trajectory. [file 4662480.f4.pdf]

Supplementary Table S2: Gene clusters expressed along the pseudotime trajectory

Gene	Clusters
AP006222.2	1
MRPL20	1
NADK	1
C1orf86	1
RER1	1
TNFRSF14	1
MMEL1	1
PGD	1
PLOD1	1
DHRS3	1
TMEM51	1
FHAD1	1
SDHB	1
IGSF21	1
CAPZB	1
MINOS1	1
PLA2G2D	1
DDOST	1
LINC00339	1
C1QA	1
C1QC	1
C1QB	1
EPHB2	1
HMGCL	1
FUCA1	1
MAN1C1	1
ZNF593	1
IFI6	1
STX12	1
ATPIF1	1
PHACTR4	1
SNHG12	1
RAB42	1
LAPTM5	1
SDC3	1
FABP3	1
COL8A2	1
LSM10	1
ZC3H12A	1
PABPC4	1
CAP1	1
PPT1	1
ZMPSTE24	1
PPCS	1
PRDX1	1

AKR1A1	1
TMEM69	1
PIK3R3	1
MKNK1	1
ELAVL4	1
TXNDC12	1
BTF3L4	1
SCP2	1
TMEM59	1
JUN	1
ATG4C	1
TCTEX1D1	1
WLS	1
PIGK	1
IFI44L	1
CTBS	1
15-Sep	1
LMO4	1
GBP4	1
ABCD3	1
SORT1	1
GSTM4	1
LAMTOR5	1
DRAM2	1
ADORA3	1
RAP1A	1
OLFML3	1
SIKE1	1
PTGFRN	1
REG4	1
FCGR1B	1
POLR3GL	1
FCGR1A	1
PLEKHO1	1
APH1A	1
C1orf54	1
CTSS	1
CTSK	1
TNFAIP8L2	1
TCHH	1
S100A13	1
SLC39A1	1
JTB	1
C1orf43	1
RP11-350G8.5	1
KRTCAP2	1
GBA	1

FAM189B	1
LAMTOR2	1
SEMA4A	1
SLC25A44	1
C1orf85	1
CD1B	1
IFI16	1
DUSP23	1
SLAMF8	1
NCSTN	1
CD84	1
ITLN1	1
FCGR3A	1
OLFML2B	1
MGST3	1
ALDH9A1	1
TMCO1	1
CREG1	1
SFT2D2	1
ATP1B1	1
SOAT1	1
IER5	1
GLUL	1
NPL	1
RGL1	1
RGS1	1
GLRX2	1
CFH	1
TMEM9	1
RNPEP	1
ADIPOR1	1
CYB5R1	1
BTG2	1
FAM72A	1
IL10	1
CR1	1
CD46	1
NENF	1
ATF3	1
NSL1	1
CENPF	1
RRP15	1
AIDA	1
TLR5	1
EPHX1	1
RAB4A	1
ARV1	1

GNPAT	1
NTPCR	1
SCCPDH	1
TMEM18	1
ADI1	1
PDIA6	1
PQLC3	1
LAPTM4A	1
RHOB	1
C2orf43	1
FKBP1B	1
DTNB	1
SLC35F6	1
ATRAID	1
MPV17	1
DPY30	1
RASGRP3	1
FEZ2	1
GALM	1
MORN2	1
AC007246.3	1
SLC8A1	1
SRBD1	1
PIGF	1
MCFD2	1
ACYP2	1
MDH1	1
CNRIP1	1
ANTXR1	1
ANXA4	1
TEX261	1
NAGK	1
SPR	1
STAMBP	1
BOLA3	1
LOXL3	1
DOK1	1
TGOLN2	1
CAPG	1
VAMP8	1
TMEM150A	1
CHMP3	1
ANKRD36C	1
DUSP2	1
LMAN2L	1
COX5B	1
COA5	1

MGAT4A	1
AC109826.1	1
SULT1C2	1
LINC00116	1
MERTK	1
TMEM87B	1
TMEM37	1
PTPN18	1
MGAT5	1
HNMT	1
RND3	1
FMNL2	1
NR4A2	1
CD302	1
SSB	1
METTL5	1
CYBRD1	1
GPR155	1
DNAJC10	1
SLC40A1	1
OSGEPL1	1
STAT1	1
SLC39A10	1
HSPE1	1
PLCL1	1
SPATS2L	1
NDUFB3	1
CD28	1
IDH1	1
RPE	1
PNKD	1
CYP27A1	1
MFF	1
PSMD1	1
ARL4C	1
BHLHE40	1
CAMK1	1
RPUSD3	1
FGD5	1
ZFYVE20	1
UBE2E2	1
SLC4A7	1
CMC1	1
TGFBR2	1
GLB1	1
CRTAP	1
ITGA9	1

ENTPD3-AS1	1
SHISA5	1
NDUFAF3	1
KLHDC8B	1
GPX1	1
RHOA	1
TCTA	1
GNAI2	1
TUSC2	1
MAPKAPK3	1
TEX264	1
ABHD14A	1
PPM1M	1
NISCH	1
STAB1	1
SMIM4	1
GLT8D1	1
TMEM110	1
CACNA2D3	1
DNASE1L3	1
SUCLG2	1
CTD-2013N24.2	1
ARL6IP5	1
FRMD4B	1
MITF	1
MTRNR2L12	1
NFKBIZ	1
ALCAM	1
ATP6V1A	1
TIMMDC1	1
ITGB5	1
CHST13	1
CHCHD6	1
PLXND1	1
ATP2C1	1
NUDT16	1
ANAPC13	1
RP11-85F14.5	1
MRAS	1
RBP1	1
HLTF	1
SUCNR1	1
SSR3	1
TIPARP	1
LXN	1
RARRES1	1
MFSD1	1

SERPINI1	1
GOLIM4	1
GPR160	1
NCEH1	1
RP11-145M9.4	1
GNB4	1
DNAJC19	1
PARL	1
ABCC5	1
ALG3	1
ETV5	1
ST6GAL1	1
LEPREL1	1
HES1	1
AC069513.4	1
TFRC	1
MFSD7	1
C4orf48	1
RGS12	1
LRPAP1	1
CYTL1	1
TBC1D14	1
SLC2A9	1
HS3ST1	1
FAM200B	1
QDPR	1
LAP3	1
SMIM20	1
PGM2	1
TBC1D1	1
TLR1	1
RBM47	1
TMEM33	1
OCIAD1	1
SRD5A3	1
PF4V1	1
NAAA	1
CXCL9	1
CXCL10	1
SCARB2	1
LINC01094	1
BMP2K	1
RASGEF1B	1
ENOPH1	1
MRPS18C	1
ABCG2	1
SNCA	1

CISD2	1
SEC24B-AS1	1
PLA2G12A	1
KIAA1109	1
RP13-884E18.2	1
NDUFC1	1
MGST2	1
CTSO	1
PDGFC	1
FAM198B	1
TMEM144	1
1-Mar	1
RWDD4	1
KIAA1430	1
CYP4V2	1
MIR4458HG	1
CTNND2	1
FAM105A	1
ANKH	1
FYB	1
DAB2	1
PTGER4	1
SEPP1	1
EMB	1
NDUFS4	1
NAIP	1
FCHO2	1
HEXB	1
IQGAP2	1
CRHBP	1
AGGF1	1
LHFPL2	1
MTRNR2L2	1
MEF2C	1
RHOBTB3	1
GLRX	1
REEP5	1
CCDC112	1
DTWD2	1
HSD17B4	1
SNX2	1
SNX24	1
PPIC	1
ACSL6	1
UQCRQ	1
TGFBI	1
EGR1	1

CD14	1
GNPDA1	1
NDFIP1	1
ABLIM3	1
CSF1R	1
GPX3	1
GM2A	1
SLC36A1	1
ATOX1	1
LARP1	1
HAVCR2	1
TTC1	1
NUDCD2	1
DOCK2	1
DUSP1	1
RPL26L1	1
ATP6V0E1	1
LMAN2	1
RGS14	1
TMED9	1
RUFY1	1
CANX	1
LTC4S	1
RNF130	1
MAPK9	1
SERPINB6	1
NQO2	1
PXDC1	1
F13A1	1
LY86	1
SSR1	1
TMEM14C	1
TMEM14B	1
MCUR1	1
CD83	1
MYLIP	1
TPMT	1
C6orf62	1
RP3-369A17.4	1
HLA-E	1
HLA-C	1
AIF1	1
HSPA1A	1
HSPA1B	1
NEU1	1
C2	1
HLA-DMB	1

HLA-DMA	1
HLA-DOA	1
WDR46	1
CUTA	1
C6orf1	1
NUDT3	1
UHRF1BP1	1
GLO1	1
TREM2	1
CUL9	1
DNPH1	1
YIPF3	1
SLC29A1	1
HSP90AB1	1
NFKBIE	1
RP11-444E17.6	1
PLA2G7	1
PAQR8	1
GCLC	1
DST	1
SLC17A5	1
ME1	1
RRAGD	1
PRDM1	1
SEC63	1
OSTM1	1
SNX3	1
CD164	1
FIG4	1
FAM26F	1
RWDD1	1
HDDC2	1
HINT3	1
ARHGAP18	1
EPB41L2	1
AKAP7	1
STX7	1
SLC18B1	1
SGK1	1
RP11-557H15.4	1
IFNGR1	1
CCDC28A	1
ABRACL	1
RP3-460G2.2	1
GPR126	1
AIG1	1
FUCA2	1

PHACTR2	1
UTRN	1
RAB32	1
SASH1	1
PCMT1	1
CCDC170	1
GTF2H5	1
TAGAP	1
SLC22A1	1
MPC1	1
RNASET2	1
FAM20C	1
TTYH3	1
KDELR2	1
ZDHHC4	1
NDUFA4	1
TMEM106B	1
MACC1	1
GPNMB	1
FAM221A	1
SKAP2	1
HIBADH	1
SCRN1	1
EEPD1	1
TMED4	1
VOPP1	1
GBAS	1
GUSB	1
LIMK1	1
NCF1	1
CCL24	1
HSPB1	1
FGL2	1
CD36	1
HGF	1
TP53TG1	1
FZD1	1
AKAP9	1
PON2	1
BRI3	1
LAMTOR4	1
GAL3ST4	1
AP1S1	1
ZNHIT1	1
RABL5	1
ARMC10	1
PSMC2	1

GPR85	1
LINC00998	1
CPED1	1
NDUFA5	1
METTL2B	1
CALU	1
ATP6V1F	1
RP11-38M8.1	1
AKR1B1	1
WDR91	1
TBXAS1	1
PARP12	1
NDUFB2	1
PDIA4	1
ATP6V0E2	1
LINC00996	1
GIMAP8	1
GIMAP4	1
GIMAP1	1
GIMAP5	1
TMEM176B	1
TMEM176A	1
CDK5	1
TMUB1	1
RHEB	1
DHRSX	1
ARSD	1
RP11-733O18.1	1
TLR7	1
TLR8	1
PIR	1
MBTPS2	1
PRDX4	1
CYBB	1
MID1IP1	1
ATP6AP2	1
GPR34	1
GPR82	1
VSIG4	1
EDA	1
COX7B	1
ATP7A	1
CYSLTR1	1
SH3BGRL	1
ARMCX1	1
NGFRAP1	1
TCEAL4	1

TCEAL3	1
TCEAL1	1
PGRMC1	1
TMEM255A	1
LAMP2	1
MCTS1	1
AIFM1	1
VMA21	1
BCAP31	1
SSR4	1
ARHGAP4	1
RENBP	1
DNASE1L1	1
ATP6AP1	1
MPP1	1
TMLHE	1
CLN8	1
CTSB	1
MSR1	1
ASAH1	1
LPL	1
GFRA2	1
DOK2	1
SORBS3	1
ADAM28	1
ADAMDEC1	1
DPYSL2	1
LEPROTL1	1
RP11-11N9.4	1
EIF4EBP1	1
HTRA4	1
ADAM9	1
SMIM19	1
GGH	1
DNAJC5B	1
TRAM1	1
TMEM70	1
LY96	1
FABP5	1
ATP6V0D2	1
WWP1	1
CPQ	1
MTDH	1
COX6C	1
ENPP2	1
RP11-489O18.1	1
SLC45A4	1

ARC	1
LY6E	1
SLC52A2	1
TONSL	1
PPAPDC2	1
BNC2	1
PTPLAD2	1
IFT74	1
ACO1	1
NDUFB6	1
CD72	1
CCDC107	1
HINT2	1
CLTA	1
FXN	1
ZFAND5	1
ALDH1A1	1
DAPK1	1
CKS2	1
GADD45G	1
NFIL3	1
FAM120A	1
SLC35D2	1
AAED1	1
TRIM14	1
CORO2A	1
GNG10	1
SLC31A2	1
SLC31A1	1
STOM	1
NDUFA8	1
PTGS1	1
NEK6	1
SLC2A8	1
ENG	1
AK1	1
DPM2	1
RP11-545E17.3	1
CCBL1	1
DOLK	1
PPP2R4	1
DPP7	1
ZMYND11	1
KLF6	1
RP11-536K7.3	1
SFMBT2	1
PHYH	1

FRMD4A	1
MRC1L1	1
SLC39A12	1
MSRB2	1
OTUD1	1
ACBD5	1
WAC-AS1	1
RP11-472N13.3	1
ARHGAP12	1
NRP1	1
ZNF32	1
CXCL12	1
NCOA4	1
RP11-556E13.1	1
CISD1	1
TMEM26	1
EGR2	1
SGPL1	1
PCBD1	1
UNC5B	1
SLC29A3	1
C10orf54	1
PSAP	1
MYOZ1	1
PLAU	1
COMTD1	1
C10orf11	1
KCNMA1	1
TMEM254	1
FAM213A	1
LIPA	1
FFAR4	1
BLNK	1
HPS1	1
PKD2L1	1
SCD	1
FAM178A	1
C10orf76	1
USMG5	1
CACUL1	1
SFXN4	1
RGS10	1
HTRA1	1
MKI67	1
MGMT	1
FUOM	1
IFITM3	1

CD151	1
TSPAN4	1
CHID1	1
AP2A2	1
IFITM10	1
CTSD	1
ASCL2	1
CD81	1
SLC22A18	1
STIM1	1
TPP1	1
PPFIBP2	1
TMEM9B	1
ADM	1
LYVE1	1
PLEKHA7	1
NUCB2	1
TMEM86A	1
CD59	1
LMO2	1
APIP	1
HSD17B12	1
EXT2	1
CKAP5	1
ACP2	1
NR1H3	1
SPI1	1
MTCH2	1
UBE2L6	1
SERPING1	1
CTNND1	1
MPEG1	1
MS4A6A	1
MS4A4A	1
MS4A7	1
MS4A14	1
CYB561A3	1
TMEM138	1
RAB3IL1	1
B3GAT3	1
C11orf83	1
TMEM179B	1
ATL3	1
RTN3	1
MARK2	1
COX8A	1
VEGFB	1

PRDX5	1
BATF2	1
ARL2	1
CCDC85B	1
YIF1A	1
ZDHHC24	1
CTSF	1
ADRBK1	1
POLD4	1
TMEM134	1
NDUFV1	1
UNC93B1	1
ALDH3B1	1
CCND1	1
LAMTOR1	1
FOLR2	1
INPPL1	1
SLCO2B1	1
TPBGL	1
ARRB1	1
ACER3	1
NDUFC2	1
ALG8	1
TMEM126A	1
CTSC	1
ALKBH8	1
ACAT1	1
SDHD	1
IL18	1
PTS	1
CADM1	1
USP2	1
ROBO3	1
SLC37A2	1
TMEM218	1
RP11-480C22.1	1
ST14	1
NINJ2	1
C12orf5	1
DYRK4	1
CD9	1
LTBR	1
TAPBPL	1
PTMS	1
CD4	1
LPCAT3	1
CD163L1	1

CD163	1
C3AR1	1
M6PR	1
A2M	1
CLEC2B	1
CREBL2	1
HEBP1	1
PLBD1	1
H2AFJ	1
DERA	1
CMAS	1
ITPR2	1
TM7SF3	1
ZCRB1	1
SLC48A1	1
TMBIM6	1
COX14	1
METTL7A	1
NR4A1	1
ITGB7	1
SMUG1	1
BLOC1S1	1
ESYT1	1
CNPY2	1
LRP1	1
PIP4K2C	1
OS9	1
TSPAN31	1
FAM19A2	1
GNS	1
TMEM19	1
PLXNC1	1
IKBIP	1
ARL1	1
CHPT1	1
GNPTAB	1
CCDC53	1
IGF1	1
HSP90B1	1
APPL2	1
CMKLR1	1
USP30-AS1	1
ANAPC7	1
VPS29	1
HVCN1	1
TMEM116	1
OAS1	1

TPCN1	1
PLBD2	1
SDS	1
SDSL	1
MLEC	1
SCARB1	1
CRYL1	1
N6AMT2	1
MRP63	1
MTIF3	1
SLC46A3	1
ALOX5AP	1
ALG5	1
NHLRC3	1
LACC1	1
TSC22D1	1
NUDT15	1
ITM2B	1
RB1	1
LPAR6	1
SETDB2	1
ARL11	1
KCTD12	1
RAP2A	1
FARP1	1
TM9SF2	1
TNFSF13B	1
RAB20	1
LAMP1	1
TMEM255B	1
RNASE6	1
RNASE1	1
DAD1	1
SLC7A7	1
LRP10	1
SLC7A8	1
DHRS4	1
DHRS4L2	1
PCK2	1
GMPR2	1
SPTSSA	1
SNX6	1
RP11-547D23.1	1
LGALS3	1
EXOC5	1
JKAMP	1
RTN1	1

SLC38A6	1
SGPP1	1
MAX	1
PIGH	1
VTI1B	1
RDH11	1
SYNJ2BP	1
ABCD4	1
NPC2	1
TMED10	1
FOS	1
ANGEL1	1
C14orf159	1
LGMN	1
ITPK1	1
IFI27	1
IFI27L2	1
EVL	1
DEGS2	1
APOPT1	1
NUDT14	1
CYFIP1	1
RP11-1008C21.1	1
SPRED1	1
PLCB2	1
RMDN3	1
GCHFR	1
SERF2	1
CASC4	1
B2M	1
TRIM69	1
GATM	1
GALK2	1
CCPG1	1
MNS1	1
TCF12	1
TPM1	1
HERC1	1
FAM96A	1
PPIB	1
SPG21	1
MTFMT	1
CLN6	1
UACA	1
THAP10	1
HEXA	1
CD276	1

CSK	1
ULK3	1
SCAMP2	1
COMMD4	1
TSPAN3	1
TBC1D2B	1
CTSH	1
TM6SF1	1
C15orf38	1
MEF2A	1
FAM195A	1
FAM173A	1
HAGHL	1
CLCN7	1
HAGH	1
NDUFB10	1
PGP	1
AMDHD2	1
TCEB2	1
HCFC1R1	1
MMP25	1
NMRAL1	1
CPPED1	1
NTAN1	1
GDE1	1
KNOP1	1
ACSM5	1
IGSF6	1
OTOA	1
EEF2K	1
HS3ST2	1
NDUFAB1	1
NUPR1	1
SULT1A1	1
QPRT	1
TMEM219	1
PYCARD	1
RP11-452L6.7	1
HEATR3	1
ADCY7	1
LPCAT2	1
AMFR	1
MT1G	1
MT1H	1
CETP	1
COQ9	1
GOT2	1

TK2	1
CMTM3	1
TMEM208	1
AGRP	1
DPEP3	1
DPEP2	1
PLA2G15	1
MAF	1
GCSH	1
HSBP1	1
IRF8	1
CYBA	1
TRAPPC2L	1
RILP	1
CLUH	1
CTNS	1
CXCL16	1
VMO1	1
SCIMP	1
TXNDC17	1
XAF1	1
RNASEK	1
CTDNEP1	1
TNFSF12	1
CD68	1
STX8	1
GAS7	1
SCO1	1
TRPV2	1
PLD6	1
PEMT	1
SLC47A1	1
ALDH3A2	1
WSB1	1
SLC46A1	1
ADAP2	1
RNF135	1
EVI2A	1
COPRS	1
CCL8	1
CCL13	1
CCL18	1
CCL3L1	1
CCL4L2	1
CISD3	1
PLXDC1	1
STARD3	1

GSDMA	1
IGFBP4	1
RAB5C	1
ATP6V0A1	1
NAGLU	1
MLX	1
COA3	1
IFI35	1
VAT1	1
G6PC3	1
GRN	1
COPZ2	1
GNGT2	1
ABI3	1
PDK2	1
MRPL27	1
ACSF2	1
SPATA20	1
ABCC3	1
SCPEP1	1
CLTC	1
VMP1	1
RP11-178C3.2	1
MRC2	1
ACE	1
CCDC47	1
MILR1	1
FAM20A	1
SSTR2	1
CD300A	1
CD300LF	1
ATP5H	1
GRB2	1
TRIM47	1
METTL23	1
SOCS3	1
TIMP2	1
LGALS3BP	1
GAA	1
EIF4A3	1
RNF213	1
BAIAP2	1
MRPL12	1
P4HB	1
SECTM1	1
COLEC12	1
RP11-838N2.4	1

RP11-760N9.1	1
OSBPL1A	1
DSC2	1
ACAA2	1
MRO	1
ME2	1
ELAC1	1
WDR7	1
NARS	1
SEC11C	1
KDSR	1
TIMM21	1
CNDP2	1
CSNK2A1	1
FAM110A	1
DDRGK1	1
C20orf194	1
SIGLEC1	1
LAMP5	1
ESF1	1
SNX5	1
MGME1	1
LINC00493	1
NAA20	1
ABHD12	1
EDEM2	1
C20orf24	1
NDRG3	1
SAMHD1	1
RPN2	1
PIGT	1
CTSA	1
PLTP	1
MMP9	1
SLC12A5	1
FAM210B	1
GNAS	1
CTSZ	1
BIRC7	1
RGS19	1
BSG	1
CFD	1
MIDN	1
C19orf24	1
NDUFS7	1
REEP6	1
UQCR11.1	1

TIMM13	1
GADD45B	1
SLC39A3	1
NCLN	1
NFIC	1
MFSD12	1
C19orf10	1
CLEC4G	1
CD209	1
CTD-2369P2.2	1
FDX1L	1
ILF3-AS1	1
ACP5	1
MAN2B1	1
DNASE2	1
CALR	1
STX10	1
IER2	1
CC2D1A	1
GIPC1	1
DNAJB1	1
TECR	1
NDUFB7	1
WIZ	1
KLF2	1
SMIM7	1
MRPL34	1
BST2	1
COLGALT1	1
JUND	1
GDF15	1
LRRC25	1
C19orf60	1
ATP13A1	1
CEBPA	1
PEPD	1
USF2	1
TMEM147	1
COX6B1	1
IGFLR1	1
TYROBP	1
ZNF566	1
CTD-2162K18.5	1
C19orf33	1
YIF1B	1
RINL	1
FCGBP	1

PLD3	1
BLVRB	1
AXL	1
AC006129.4	1
RABAC1	1
ETHE1	1
APOE	1
APOC1	1
APOC2	1
ERCC2	1
FOSB	1
PPM1N	1
CALM3	1
AP2S1	1
TMEM160	1
SAE1	1
SEPW1	1
KDELR1	1
CA11	1
HSD17B14	1
NUCB1	1
DHDH	1
FTL	1
FCGRT	1
EMC10	1
CLEC11A	1
SIGLEC7	1
CD33	1
SIGLEC10	1
SIGLEC8	1
SIGLEC12	1
FPR3	1
ZNF331	1
NDUFA3	1
TFPT	1
CTD-2337J16.1	1
LILRB5	1
LAIR1	1
LILRB4	1
EPN1	1
CECR1	1
COMT	1
DGCR6L	1
SNAP29	1
XXbac-B135H6.15	1
TPST2	1
CRYBB1	1

AP1B1	1
UQCR10	1
TCN2	1
PIK3IP1	1
YWHAH	1
APOL6	1
FOXRED2	1
NCF4	1
CYTH4	1
CDC42EP1	1
MAFF	1
RPS19BP1	1
NAGA	1
NFAM1	1
TTLL12	1
PARVB	1
PRR5	1
KIAA0930	1
TTC38	1
CRELD2	1
ARSA	1
ATP5J	1
IFNAR2	1
RCAN1	1
CBR1	1
PIGP	1
PSMG1	1
MX1	1
NDUFV3	1
SIK1	1
C21orf33	1
PTTG1IP	1
ITGB2	1
COL6A1	1
COL6A2	1
MT-ND2	1
MT-CO2	1
MT-ATP6	1
MT-CO3	1
MT-ND3	1
MT-ND4	1
MT-CYB	1
ISG15	2
MXRA8	2
MMP23B	2
ERRFI1	2
TNFRSF8	2

TNFRSF1B	2
ZBTB17	2
CDA	2
CLIC4	2
C1orf63	2
TMEM50A	2
FGR	2
TXLNA	2
C1orf122	2
MTF1	2
MFSD2A	2
SSBP3	2
PPAP2B	2
JAK1	2
AK4	2
TMED5	2
F3	2
RP11-284N8.3	2
CHI3L2	2
ZNF697	2
NOTCH2	2
NBPF9	2
PDE4DIP	2
NBPF10	2
RP11-277L2.5	2
MCL1	2
S100A9	2
S100A12	2
S100A8	2
RAB13	2
IL6R	2
ETV3L	2
SLAMF9	2
SLAMF1	2
CD48	2
FCER1G	2
FCGR2B	2
FCRLB	2
MPZL1	2
SELE	2
QSOX1	2
NCF2	2
IVNS1ABP	2
PTGS2	2
PTPN7	2
PPP1R15B	2
IL24	2

LAMB3	2
RP1-28O10.1	2
G0S2	2
SLC30A1	2
RP11-400N13.3	2
WDR26	2
GUK1	2
KMO	2
FOSL2	2
CRIM1	2
QPCT	2
CDC42EP3	2
CYP1B1	2
RHOQ	2
RAB1A	2
PLEK	2
MXD1	2
HK2	2
GNLY	2
IL1R1	2
IL1RL2	2
LIMS1	2
SOWAHC	2
IL1A	2
IL1B	2
IL36G	2
IL1RN	2
MARCO	2
RAB3GAP1	2
KYNU	2
ZEB2	2
TNFAIP6	2
ACVR1	2
TANK	2
AC093818.1	2
MLTK	2
SESTD1	2
NAB1	2
NABP1	2
HSPD1	2
BZW1	2
RP11-469M7.1	2
TNS1	2
SLC11A1	2
ACSL3	2
CCL20	2
PID1	2

DNER	2
D2HGDH	2
NEU4	2
ARL8B	2
IRAK2	2
SLC6A6	2
SH3BP5	2
EAF1	2
UBE2E1	2
EIF1B	2
CTNNB1	2
CDCP1	2
TMEM158	2
CCR5	2
CCRL2	2
RYBP	2
PPP4R2	2
GBE1	2
CGGBP1	2
PIK3CB	2
ZBTB38	2
GK5	2
PLSCR1	2
TM4SF1	2
PTX3	2
TNIK	2
PLD1	2
PP13439	2
FNDC3B	2
ATP11B	2
B3GNT5	2
BCL6	2
IL1RAP	2
ATP13A3	2
LINC00884	2
ZDHHC19	2
TCTEX1D2	2
TM4SF19.1	2
LRCH3	2
N4BP2	2
RHOH	2
CHIC2	2
IL8	2
CXCL1	2
PPBP	2
CXCL5	2
CXCL3	2

CXCL2	2
EREG	2
AREG	2
CXCL11	2
PRDM8	2
HPSE	2
SPP1	2
SLC39A8	2
NFKB1	2
C4orf3	2
TNIP3	2
ANXA5	2
SLC7A11	2
TLR2	2
RAPGEF2	2
DDX60L	2
ACSL1	2
RP11-701P16.5	2
RP11-462G22.1	2
LPCAT1	2
MYO10	2
BASP1	2
ELOVL7	2
ZFYVE16	2
VCAN	2
MEF2C-AS1	2
LUCAT1	2
ELL2	2
PRR16	2
SEC24A	2
CYSTM1	2
HBEGF	2
SPINK1	2
TNIP1	2
CTB-114C7.4	2
STK10	2
UBTD2	2
ERGIC1	2
PDLIM7	2
DOK3	2
HIVEP1	2
EDN1	2
PHACTR1	2
TBC1D7	2
RNF144B	2
HIST1H1C	2
PPP1R11	2

IER3	2
BRPF3	2
TREM1	2
VEGFA	2
C6orf223	2
NT5E	2
AKIRIN2	2
PNRC1	2
SLC16A10	2
RP11-367G18.1	2
MARCKS	2
DSE	2
RP1-93H18.7	2
RP3-325F22.5	2
HIVEP2	2
TAB2	2
SOD2	2
WTAP	2
RP1-249F5.3	2
MAP3K4	2
PHF10	2
DLL1	2
AC147651.4	2
ZFAND2A	2
GNA12	2
TWISTNB	2
CTA-293F17.1	2
ITGB8	2
IL6	2
PPP1R17	2
INHBA	2
PURB	2
HUS1	2
UPP1	2
SEC61G	2
RABGEF1	2
PTPN12	2
AC002456.2	2
PILRA	2
MUC17	2
SERPINE1	2
NAMPT	2
DOCK4	2
ZNF277	2
MET	2
HILPDA	2
STRIP2	2

MIR29A	2
LINC-PINT	2
KDM7A	2
JHDM1D-AS1	2
MGAM	2
CLEC5A	2
AC093673.5	2
PRKAG2	2
INSIG1	2
PLCXD1	2
IL3RA	2
CD99	2
GS1-600G8.5	2
SMS	2
SAT1	2
GK	2
RPGR	2
TIMP1	2
HUWE1	2
IL2RG	2
PGK1	2
ACSL4	2
MST4	2
RAP2C	2
MAMLD1	2
IRAK1	2
TKTL1	2
FLNA	2
DEFB1	2
RP11-1080G15.1	2
EGR3	2
SLC25A37	2
BNIP3L	2
AGPAT6	2
SDCBP	2
NSMAF	2
MSC	2
HEY1	2
PAG1	2
RIPK2	2
NBN	2
SDC2	2
KB-1507C5.4	2
ASAP1	2
NDRG1	2
RP11-629O1.2	2
GRINA	2

CTD-3065J16.6	2
CD274	2
MOB3B	2
B4GALT1	2
UBAP2	2
CTSL	2
S1PR3	2
NINJ1	2
MSANTD3	2
SLC44A1	2
TNFSF15	2
TNFSF8	2
HSPA5	2
FAM129B	2
SLC2A6	2
OPTN	2
ITGB1	2
CSGALNACT2	2
HNRNPF	2
SGMS1	2
RP11-120C12.3	2
HERC4	2
SRGN	2
SAR1A	2
P4HA1	2
ZMIZ1-AS1	2
PPIF	2
LIPN	2
PIK3AP1	2
ENTPD7	2
GSTO1	2
ITPRIP	2
BBIP1	2
ACSL5	2
BNIP3	2
MIR210HG	2
PHLDA2	2
NRIP3	2
DENND5A	2
CD44	2
FTH1	2
EHD1	2
SERPINH1	2
SLC36A4	2
MMP10	2
MMP1	2
CASP4	2

CASP1	2
CARD16	2
RDX	2
TBCEL	2
GAPDH	2
TPI1	2
SLC2A3	2
CLEC4D	2
CLEC4E	2
RP11-20D14.6	2
AC091814.2	2
CLEC12A	2
EMP1	2
BCAT1	2
RP11-662I13.2	2
DAZAP2	2
GPR84	2
ITGA5	2
MUCL1	2
MMP19	2
IL23A	2
MON2	2
TBK1	2
IRAK3	2
PHLDA1	2
OSBPL8	2
DUSP6	2
ATP2B1	2
LINC00936	2
C12orf79	2
BTG1	2
RP11-796E2.4	2
CRADD	2
DRAM1	2
CKAP4	2
MED13L	2
WSB2	2
OASL	2
HCAR2	2
RILPL2	2
RP11-955H22.1	2
GJB2	2
SPATA13	2
USP12	2
FLT1	2
CCNA1	2
RGCC	2

LINC01050	2
LCP1	2
FNDC3A	2
FAM124A	2
IRG1	2
DNAJC3	2
PNP	2
RN7SL1	2
C14orf182	2
GNG2	2
PTGER2	2
ERO1L	2
RP11-841O20.2	2
GCH1	2
RP11-349A22.5	2
PRKCH	2
RP11-47I22.2	2
HIF1A	2
HIF1A-AS2	2
SNAPC1	2
ACTN1	2
RP4-647C14.3	2
NUMB	2
RIN3	2
SERPINA1	2
HSP90AA1	2
TDRD9	2
SCG5	2
C15orf48	2
RP11-519G16.3	2
AQP9	2
RAB8B	2
CA12	2
MAP2K1	2
MTHFS	2
ST20	2
BCL2A1	2
ABHD17C	2
KIAA1199	2
MESDC1	2
RHCG	2
ANPEP	2
IQGAP1	2
ARRDC4	2
LITAF	2
DCUN1D3	2
METTL9	2

ALDOA	2
PPP4C	2
ZNF646	2
ITGAX	2
ZNF720	2
CES1	2
MT2A	2
MT1F	2
GPR97	2
KIFC3	2
HP	2
DNAAF1	2
SLC43A2	2
MIR22HG	2
CHRNE	2
MED31	2
HS3ST3B1	2
MAP2K3	2
SDF2	2
SEZ6	2
CPD	2
CCL2	2
CCL23	2
CCL3	2
CCL4	2
CCL3L3	2
CCL4L1	2
CTB-58E17.1	2
CSF3	2
UBE2Z	2
KAT7	2
MED13	2
CD300E	2
H3F3B	2
SPHK1	2
RP11-806H10.4	2
DNAH17	2
CYTH1	2
C1QTNF1	2
AC144831.1	2
DLGAP1-AS1	2
EPB41L3	2
PSTPIP2	2
RP11-15F12.1	2
SERPINB2	2
SERPINB8	2
SMOX	2

RP4-779E11.3	2
CD93	2
MAP1LC3A	2
SRC	2
SDC4	2
B4GALT5	2
RP11-290F20.3	2
SLCO4A1	2
LINC00659	2
DOT1L	2
SEMA6B	2
TNFSF14	2
STXBP2	2
RETN	2
C19orf59	2
ANGPTL4	2
ZSWIM4	2
EMR3	2
EMR2	2
MAP1S	2
FFAR2	2
HCST	2
NFKBIB	2
IRGQ	2
PLAUR	2
MEIS3	2
EMP3	2
SIGLEC14	2
HAS1	2
FPR2	2
OSCAR	2
LILRA2	2
FCAR	2
CTB-61M7.2	2
ZNF787	2
MIF	2
GGT5	2
XBP1	2
LIMK2	2
TOM1	2
RAC2	2
APOBEC3B	2
MEI1	2
ARFGAP3	2
AF165138.7	2
NRIP1	2
BTG3	2

MIR155HG	2
MAP3K7CL	2
BACH1	2
ETS2	2
CSTB	2
RRP1	2
TNFRSF18	3
ENO1	3
GPR157	3
ECE1	3
MARCKSL1	3
SFPQ	3
SLC2A1	3
GADD45A	3
DNTTIP2	3
CD58	3
MTMR11	3
S100A10	3
RPS27	3
ARHGEF2	3
TAGLN2	3
UAP1	3
LAD1	3
CD55	3
H3F3A	3
RP11-295G20.2	3
LYST	3
ADSS	3
NLRP3	3
ID2	3
VRK2	3
LINC00152	3
MAP4K4	3
MIR4435-1HG	3
AC017002.1	3
CYTIP	3
LY75	3
SP3	3
CFLAR	3
ACKR3	3
RAMP1	3
ANKRD28	3
LRRFIP2	3
OXSR1	3
SELK	3
PROK2	3
LINC00877	3

CD200	3
POGLUT1	3
CSTA	3
MSL2	3
ATP1B3	3
CHST2	3
SIAH2	3
SKIL	3
LAMP3	3
IGF2BP2	3
MFI2	3
RPL35A	3
TNIP2	3
LIMCH1	3
GRSF1	3
ANTXR2	3
PLAC8	3
DAPP1	3
PPP3CA	3
TET2	3
SGMS2	3
MED10	3
SUB1	3
IL7R	3
GPBP1	3
MAST4	3
STARD4	3
TNFAIP8	3
LMNB1	3
PPP2CA	3
TAF7	3
NR3C1	3
SLC26A2	3
ANXA6	3
ADAM19	3
EBF1	3
RNF145	3
IRF4	3
SERPINB1	3
SERPINB9	3
JARID2	3
HLA-DOB	3
KCTD20	3
CDKN1A	3
EEF1A1	3
FSCN1	3
TSPAN13	3

TRA2A	3
HOTAIRM1	3
MYO1G	3
TPST1	3
AC003092.1	3
ACHE	3
HIPK2	3
BRAF	3
AP1S2	3
SYAP1	3
ACOT9	3
CHST7	3
SYN1	3
CFP	3
UXT	3
PLP2	3
HNRNPH2	3
EMD	3
RPL10	3
CLU	3
DUSP4	3
IDO1	3
YWHAZ	3
MTSS1	3
CASC7	3
CCL19	3
GLIPR2	3
BX255923.3	3
SEC61B	3
TXN	3
TRAF1	3
FNBP1	3
FCN1	3
VIM-AS1	3
VIM	3
MAP3K8	3
CREM	3
PPA1	3
NFKB2	3
DUSP5	3
ADAM8	3
IRF7	3
LSP1	3
LDHA	3
LDLRAD3	3
C11orf96	3
NRXN2	3

FAU	3
MALAT1	3
CLCF1	3
BIRC3	3
OLR1	3
BCL2L14	3
PLEKHA5	3
VDR	3
PFDN5	3
MYL6	3
RAP1B	3
SOCS2	3
ELK3	3
SLC41A2	3
NAA25	3
ANKLE2	3
TPT1	3
TBC1D4	3
CTSG	3
GZMB	3
BAZ1A	3
RPL36AL	3
SRSF5	3
SIPA1L1	3
WARS	3
GPR132	3
CRIP1	3
THBS1	3
PKM	3
ISG20	3
TPSAB1	3
USP7	3
TVP23A	3
NDE1	3
CCL22	3
CCL17	3
CMTM2	3
NFAT5	3
GABARAPL2	3
SLC7A5	3
ANKRD11	3
C17orf107	3
TMEM107	3
ALDOC	3
CCR7	3
EIF1	3
CTD-2020K17.1	3

DDX5	3
ARHGDIA	3
MYL12B	3
ALPK2	3
MALT1	3
HMSD	3
SNRPB	3
INSM1	3
THBD	3
CST7	3
CHMP4B	3
LINC00657	3
CTNNBL1	3
TGM2	3
PTPN1	3
ADNP	3
PMEPA1	3
MOB3A	3
GNA15	3
EBI3	3
CD70	3
TRIP10	3
CDC37	3
PDE4A	3
CHERP	3
UBA52	3
PBX4	3
GRAMD1A	3
PPP1R14A	3
NCCRP1	3
RPS16	3
B9D2	3
LYPD3	3
RELB	3
PTGIR	3
FPR1	3
VSTM1	3
BID	3
IGLL5	3
UPB1	3
NEFH	3
APOBEC3A	3
SAMSN1	3
RUNX1	3
RPL22	4
UTS2	4
PARK7	4

CTNNBIP1	4
SRM	4
FBXO6	4
RCC2	4
MRTO4	4
AKR7A2	4
SRRM1	4
STMN1	4
HMGN2	4
NUDC	4
THEMIS2	4
PSMB2	4
MRPS15	4
NDUFS5	4
YBX1	4
ERI3	4
UQCRH	4
CMPK1	4
HSPB11	4
ITGB3BP	4
SERBP1	4
TYW3	4
ACADM	4
PRKACB	4
PSMA5	4
ATP5F1	4
C1orf162	4
MRPS21	4
SCNM1	4
MRPL9	4
SNAPIN	4
PMVK	4
CKS1B	4
SCAMP3	4
LMNA	4
CCT3	4
APOA1BP	4
MRPL24	4
CD1C	4
MNDA	4
UFC1	4
NDUFS2	4
HSD17B7	4
MPC2	4
MRPS14	4
ARPC5	4
RGS18	4

RGS2	4
SNRPE	4
NUCKS1	4
TRAF3IP3	4
LYPLAL1	4
PARP1	4
MRPL55	4
RNF187	4
IRF2BP2	4
HNRNPU	4
ACP1	4
RNASEH1	4
DDX1	4
HADHB	4
SNX17	4
SRSF7	4
ZFP36L2	4
TTC7A	4
CALM2	4
NFU1	4
FAM136A	4
PRADC1	4
TPRKB	4
MOB1A	4
AUP1	4
POLE4	4
SUCLG1	4
VAMP5	4
PTCD3	4
EIF5B	4
RANBP2	4
CHCHD5	4
DDX18	4
DBI	4
GYPC	4
MZT2B	4
IMP4	4
ARHGAP15	4
ARL5A	4
PSMD14	4
WIPF1	4
ATP5G3	4
ITGA4	4
PPIL3	4
EEF1B2	4
AC079767.4	4
ARPC2	4

STK16	4
SP110	4
PTMA	4
UBE2F	4
MYEOV2	4
PPP1R7	4
HDLBP	4
SEPT2	4
DTYMK	4
MTMR14	4
ARPC4	4
LSM3	4
MRPS25	4
SATB1	4
CSRNP1	4
RPSA	4
ZDHHC3	4
UQCRC1	4
APEH	4
CYB561D2	4
ABHD14B	4
TWF2	4
SPCS1	4
TKT	4
APPL1	4
CMSS1	4
FILIP1L	4
NDUFB4	4
HCLS1	4
CNBP	4
MRPL3	4
SRPRB	4
GYG1	4
SELT	4
P2RY13	4
RAP2B	4
RPL22L1	4
NDUFB5	4
AP2M1	4
PSMD2	4
EIF4A2	4
ATP5I	4
RBPJ	4
COMMD8	4
IGJ	4
PYURF	4
HADH	4

OSTC	4
SNHG8	4
SCOC	4
PRMT10	4
RPS3A	4
DCTD	4
PDCD6	4
NDUFS6	4
DHX29	4
TBCA	4
JMY	4
COX7C	4
COMMD10	4
HINT1	4
DDX46	4
H2AFY	4
SIL1	4
NDUFA2	4
ZMAT2	4
LARS	4
CD74	4
PTTG1	4
NPM1	4
HIGD2A	4
CLTB	4
NHP2	4
MRS2	4
ACOT13	4
HIST1H4C	4
MRPS18B	4
TUBB	4
LST1	4
CSNK2B	4
LSM2	4
C6orf48	4
HLA-DRA	4
HLA-DRB5	4
HLA-DRB1	4
HLA-DQA1	4
HLA-DQB1	4
HLA-DQA2	4
HLA-DQB2	4
PSMB9	4
HLA-DPA1	4
HLA-DPB1	4
PFDN6	4
UQCC2	4

RPS10	4
SNRPC	4
DEF6	4
RPL10A	4
FGD2	4
PIM1	4
CCDC167	4
CNPY3	4
HMGN3	4
NDUFAF4	4
GTF3C6	4
IL22RA2	4
SF3B5	4
MRPL18	4
C7orf50	4
NUDT1	4
C1GALT1	4
RPA3	4
AHR	4
TOMM7	4
CYCS	4
CPVL	4
GGCT	4
LSM5	4
AOAH	4
MRPL32	4
COA1	4
BLVRA	4
PPIA	4
H2AFV	4
CCT6A	4
SUMF2	4
WBSCR22	4
RFC2	4
MDH2	4
TMEM243	4
GATAD1	4
ARPC1B	4
ATP5J2	4
COPS6	4
FIS1	4
IFRD1	4
C7orf55	4
MRPS33	4
SSBP1	4
GSTK1	4
GIMAP7	4

GIMAP6	4
TMSB4X	4
EIF1AX	4
NDUFB11	4
HSD17B10	4
MAGEH1	4
PDZD11	4
XIST	4
BTK	4
BEX4	4
TCEAL8	4
TSC22D3	4
SLC25A5	4
NDUFA1	4
HTATSF1	4
HMGB3	4
IDH3G	4
NAA10	4
LAGE3	4
MSRA	4
TMEM66	4
TM2D2	4
VDAC3	4
CEBPD	4
PRKDC	4
LYPLA1	4
MRPL15	4
TPD52	4
C8orf59	4
CPNE3	4
DECR1	4
UQCRB	4
ZNF706	4
MRPL13	4
FAM49B	4
PSCA	4
GSDMD	4
NAPRT1	4
CYC1	4
TMEM261	4
BAG1	4
GALT	4
ANP32B	4
SMC2	4
KLF4	4
GSN	4
MRRF	4

RPL35	4
MAPKAP1	4
RPL12	4
C9orf16	4
COQ4	4
ZDHHC12	4
C9orf114	4
TOR1A	4
SURF1	4
UBAC1	4
CARD9	4
TMEM141	4
PHPT1	4
FBXW5	4
C9orf142	4
MRPL41	4
IDI1	4
GDI2	4
ATP5C1	4
NUDT5	4
RSU1	4
APBB1IP	4
RASSF4	4
ALOX5	4
TSPAN15	4
PALD1	4
MRPS16	4
CHCHD1	4
ADK	4
GLUD1	4
EXOC6	4
ENTPD1	4
EXOSC1	4
BLOC1S2	4
NDUFB8.1	4
NPM3	4
FBXL15	4
PRDX3	4
UROS	4
GLRX3	4
ECHS1	4
RNH1	4
TALDO1	4
POLR2L	4
MRPL23	4
MRPL17	4
RPL27A	4

RNF141	4
PSMA1	4
CAT	4
COMMD9	4
PSMC3	4
NDUFS3	4
PTPMT1	4
TIMM10	4
C11orf31	4
TMEM109	4
DDB1	4
ASRGL1	4
POLR2G	4
RARRES3	4
DNAJC4	4
TRMT112	4
RNASEH2C	4
FIBP	4
DRAP1	4
BANF1	4
MRPL11	4
CORO1B	4
GSTP1	4
NDUFS8	4
COA4	4
UCP2	4
CLNS1A	4
PRCP	4
TMEM126B	4
C11orf73	4
TAF1D	4
TMEM123	4
TIMM8B	4
REXO2	4
PAFAH1B2	4
AMICA1	4
ATP5L	4
H2AFX	4
UBASH3B	4
HSPA8	4
THYN1	4
RAD51AP1	4
NDUFA9	4
MRPL51	4
LAG3	4
PTPN6	4
ARHGDIB	4

LDHB	4
LYRM5	4
FGFR1OP2	4
MRPS35	4
PRKAG1	4
TUBA1B	4
LIMA1	4
BIN2	4
PRR13	4
ATP5G2	4
COPZ1	4
ZNF385A	4
GTSF1	4
PA2G4	4
MYL6B	4
ATP5B	4
PTGES3	4
SHMT2	4
STAC3	4
CDK4	4
LYZ	4
RP11-1143G9.4	4
CCT2	4
GLIPR1	4
NAP1L1	4
NDUFA12	4
METAP2	4
SNRPF	4
LTA4H	4
ISCU	4
ARPC3	4
GPN3	4
PPP1CC	4
ERP29	4
PEBP1	4
RPLP0	4
COX6A1	4
ANAPC5	4
ARL6IP4	4
TMED2	4
PXMP2	4
SAP18	4
GTF3A	4
HMGB1	4
EPSTI1	4
DNAJC15	4
ESD	4

EBPL	4
SPRYD7	4
RNASEH2B	4
VPS36	4
GPR183	4
GAS6	4
OSGEP	4
APEX1	4
RNASE2	4
NDRG2	4
OXA1L	4
HAUS4	4
PSMB5	4
PSME2	4
NEDD8	4
FKBP3	4
C14orf166	4
ACTR10	4
TIMM9	4
CHURC1	4
ZFP36L1	4
COX16	4
SLIRP	4
FOXN3	4
NDUFB1	4
C14orf2	4
PLD4	4
EMC4	4
SRP14	4
DUT	4
EID1	4
GTF2A2	4
RPL4	4
UBL7	4
COX5A	4
IMP3	4
WDR61	4
TMED3	4
SEC11A	4
IDH2	4
FES	4
WASH4P	4
POLR3K	4
SNRNP25	4
MPG	4
NME4	4
C16orf13	4

NME3	4
MRPS34	4
MLST8	4
FLYWCH2	4
THOC6	4
CORO7	4
HMOX2	4
NAGPA	4
EMP2	4
UQCRC2	4
GGA2	4
TUFM	4
CORO1A	4
DCTPP1	4
CHD9	4
HERPUD1	4
CIAPIN1	4
CKLF	4
NAE1	4
COTL1	4
COX4I1	4
RNF166	4
APRT	4
SERPINF1	4
TAX1BP3	4
PSMB6	4
SLC25A11	4
PFN1	4
RPAIN	4
C1QBP	4
CLEC10A	4
ASGR1	4
TMEM256	4
ZBTB4	4
LSMD1	4
RANGRF	4
ZSWIM7	4
LGALS9	4
EVI2B	4
UTP6	4
VPS25	4
SLC25A39	4
ATP5G1	4
PHB	4
NME1	4
MMD	4
SKA2	4

DCAF7	4
PSMC5	4
GNA13	4
PRKCA	4
HN1	4
MRPS7	4
MIF4GD	4
SLC25A19	4
GALK1	4
SYNGR2	4
C17orf89	4
ANAPC11	4
STRA13	4
DCXR	4
GPS1	4
CCDC57	4
OGFOD3	4
PSMG2	4
TPGS2	4
ATP5A1	4
IER3IP1.1	4
CYB5A	4
TXNL4A	4
PSMF1	4
ITPA	4
C20orf27	4
TMEM230	4
PCNA	4
DSTN	4
CST3	4
APMAP	4
ACSS1	4
COMMD7	4
AHCY	4
DYNLRB1	4
ERGIC3	4
BLCAP	4
CD40	4
PFDN4	4
ATP5E	4
PSMA7	4
PPDPF	4
ATP5D	4
MKNK2	4
PLEKHJ1	4
C19orf70	4
NDUFA11	4

ALKBH7	4
2-Mar	4
MYO1F	4
EIF3G	4
DNMT1	4
MRPL4	4
WDR83OS	4
JUNB	4
PRDX2	4
GADD45GIP1	4
C19orf53	4
PKN1	4
PGLS	4
CCDC124	4
IFI30	4
LSM4	4
NDUFA13	4
URI1	4
FXYD5	4
POLR2I	4
SPINT2	4
KCNK6	4
EIF3K	4
ECH1	4
MRPS12	4
ZFP36	4
EXOSC5	4
RPS19	4
PAFAH1B3	4
ZNF428	4
TRAPPC6A	4
BAX	4
CD37	4
ALDH16A1	4
ETFB	4
UBE2S	4
ISOC2	4
ZNF524	4
RPS5	4
MRPL40	4
TANGO2	4
RANBP1	4
CHCHD10	4
SNRPD3	4
RTCB	4
TXN2	4
LGALS2	4

POLR2F	4
TOMM22	4
APOBEC3C	4
RPL3	4
TNRC6B	4
ADSL	4
ST13	4
RBX1	4
NHP2L1	4
SMDT1	4
NDUFA6	4
RRP7A	4
TSPO	4
SAMM50	4
PARVG	4
DENND6B	4
TYMP	4
CCT8	4
SOD1	4
ATP5O	4
PFKL	4
SUMO3	4
YBEY	4
S100B	4
PRMT2	4
